# Occurrence and risks of antibiotics in an urban river in northeastern Tibetan Plateau

**DOI:** 10.1038/s41598-020-77152-5

**Published:** 2020-11-18

**Authors:** Yuzhu Kuang, Xiaoyu Guo, Jingrun Hu, Si Li, Ruijie Zhang, Qiang Gao, Xi Yang, Qian Chen, Weiling Sun

**Affiliations:** 1 College of Resources Environment and Tourism, Capital Normal University, Beijing, 100048 China; 2 College of Environmental Sciences and Engineering, Peking University, State Environmental Protection Key Laboratory of All Materials Flux in River Ecosystems, The Key Laboratory of Water and Sediment Sciences, Ministry of Education, International Joint Laboratory for Regional Pollution Control, Ministry of Education, Beijing, 100871 China; 3 Beijing Key Laboratory of Farmland Soil Pollution Prevention and Remediation, College of Resources and Environmental Sciences, China Agricultural University, Beijing, 100193 China; 4 State Key Laboratory of Plateau Ecology and Agriculture, Qinghai University, Xining, 810016 China

**Keywords:** Environmental sciences, Natural hazards

## Abstract

There is a dearth of information on the occurrence and risks of antibiotics in the urban rivers from plateau areas. This study investigated 83 antibiotics in water and sediments of an urban river and effluents of sewage treatment plants (E-STPs) in Xining, Qinghai (northeastern Tibetan Plateau). Fifty-three antibiotics were detected, and the concentrations of individual antibiotics varied in the range of undetected (ND)-552 ng/L in water, ND-164 ng/g in sediments, and ND-3821 ng/L in E-STPs. Seasonal differences of antibiotic concentrations were significant for water samples (*p* < 0.05) but insignificant for sediments (*p* > 0.05). In urban area, E-STP is the main source of antibiotics in the river, while runoff from manured cropland contributes partially to antibiotics in the river in the suburban area. The antibiotic compositions in water were different from those in sediments, but were similar to those in E-STPs. Notably, because of strong solar radiation and long sunshine hours in the plateau area, low levels of quinolones, which are sensitive to photolysis, were observed in river water. Moreover, norfloxacin and enrofloxacin, observed in urban river from other regions of China, were not detected in the Huangshui River water. The occurrence of ofloxacin, erythromycin, roxithromycin, clarithromycin, and trimethoprim in E-STPs may induce a possible risk to antibiotic resistance evolution. Trimethoprim, anhydroerythromycin, sulfamethoxazole, sulfapyridine, and clindamycin in river water could pose low to medium risks to aquatic organisms. Further investigation on the occurrence and distribution of antibiotic resistance genes in the Huangshui River is urgently needed.

## Introduction

Antibiotics are primarily used to treat and prevent human and animal diseases, as well as growth promoters and feed efficiency improvers in agriculture and animal husbandry^1,2^. China is the largest producer and consumer of antibiotics in the world^3^. The percentage of antibiotics used for outpatients in China (50.3%) exceeded the recommended level (< 30%) by World Health Organization (WHO), and was more than three times as much as that in the USA (15.3%)^4^. The overuse of antibiotics induced their massive release into receiving waters, and thus they have been detected worldwide in surface waters^5,6^. Antibiotics in aquatic environment may change microbial communities, affect aquatic organisms, fuel the evolution and spread of antibiotic resistance genes (ARGs), and threaten human health^7,8^. Therefore, antibiotics in the aquatic environment have become a rising global concern^7^.

The high population density in cities lead to large consumption and emission of antibiotics. Sewage, hospitals, livestock and poultry farms, and aquaculture are important pollution sources of antibiotics^9^. Besides domestic sewage, industrial and hospital wastewater are also discharged into sewage treatment plants (STPs)^10^. As a pool of antibiotics, the effluent of STPs (E-STPs) showed relatively high levels of antibiotics, up to several µg/L, because of their incomplete removal by engineered biological treatment systems^9–12^. Urban rivers are the major acceptor of E-STPs, so they suffered from severe pollution of antibiotics^10,13,14^. For example, ciprofloxacin (CIP) and sulfamethoxazole (SMX) demonstrated about tenfold higher concentrations in downstream of the STP discharge than those in upstream samples^10^. The concentrations of individual antibiotics could be up to 2702 ng/L and 449 ng/g in urban river water and sediments, respectively, heavily polluted by domestic sewage^13^.

Xining is the capital of Qinghai province, with a total population of 2.355 million. It is located in the climatically sensitive semiarid zone, northeastern Tibetan Plateau, with an average altitude of 2275 m. Its typical climate is low pressure, long sunshine hours, strong solar radiation, and large difference in temperatures between day and night^15^. The average annual precipitation is about 400 mm, and 70% of the annual precipitation occurs from June to September. The annual average temperature is 3.2 °C. The Huangshui River flows through Xining city, where all the E-STPs discharged into this river (Figure S1). Despite numerous studies investigating antibiotics in the urban rivers in large and medium-sized cities^6,10,13,14^, the research on the distribution and ecological risks of antibiotics in the urban rivers from the ecologically vulnerable area, Qinghai-Tibet Plateau, is scarce.

This study investigated the occurrence of 83 antibiotics in water and sediments of the Huangshui River, and E-STPs discharged into the river. The objects of this study are (1) to investigate the occurrence and spatiotemporal distribution of antibiotics in the Huangshui River and E-STPs, (2) to compare the regional differences of antibiotic concentrations in urban rivers of China, and (3) to evaluate the possible risk of antimicrobial resistance development and potential ecological risks of antibiotics in the Huangshui River. The results provide useful information on the management of antibiotic pollution in urban rivers.

## Materials and methods

### Chemicals and reagents

Eighty-three antibiotics, covering 22 sulphonamides (SAs), 10 macrolides (MLs), 13 tetracyclines (TCs), 16 quinolones (QNs), 5 polyethers (PEs), 15 β-lactams (β-Ls), and 2 lincosamides (LMs), were quantified. The standard solutions of antibiotics were purchased from A Chemtek Inc. (Tianjin, China). The properties of the target antibiotics are given in Table S1. Isotopically labelled internal standards, which are listed in Table S1, were obtained from J&K Chemistry (Beijing, China). Chemical reagents such as citric acid and Na_2_EDTA were of analytical grade. Methanol and formic acid were of HPLC grade.

### Sample collection

**Figure 1:**
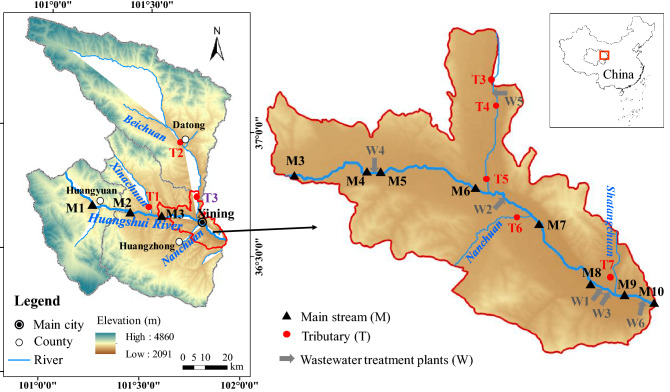
Sampling sites of the Huangshui River in Xining city. Maps created using ArcGIS 10.2‒ https://developers.arcgis.com; Image from https://www.gscloud.cn.

Water and sediment samples of the Huangshui River and water samples of E-STPs were collected in July, 2018 (wet season) and April, 2019 (dry season). The sampling sites are shown in Fig. 1. A total of 14 sampling sites were chosen in wet season, including 7 sampling sites in the mainstream (M1, M2, M3, M5, M6, M7, and M10) and 5 sampling sites in tributaries (T1, T2, T5, T6, and T7) of the Huangshui River, and 2 E-STPs (W2 and W3). When sampling in dry season, E-STPs from W1−W6 were collected to compare the antibiotic concentrations in different E-STPs. All E-STPs were discharged into the mainstream of the Huangshui River except that E-STPs from W5 were discharged into the tributary of the Huangshui River (Fig. 1). To evaluate the influence of E-STPs on the river, a total of 23 sampling sites were selected in dry season, including 10 sampling sites in the mainstream (M1−M10) and 7 sampling sites in tributaries (T1–T7) of the Huangshui River, and 6 E-STPs (W1−W6).

The water and sediment samples were collected using the same method described in our previous studies^16,17^. The collected water and sediment samples were stored in 4.5 L glass bottles and aluminum boxes, respectively. All samples were transported immediately to laboratory in an ice box, and pretreated within 24 h.

### Sample pretreatment and analysis

Water and sediment samples were pretreated according to the protocol described in our previous studies^5,17^. Briefly, water samples (2.0 L) were filtrated through glass fiber filters (0.7 μm, Whatman, UK) and concentrated using solid-phase extraction (SPE) (Oasis HLB, 500 mg, 6 mL, Waters, USA). Freeze-dried sediment samples (5.0 g) were extracted by ultrasonication and SPE sequentially. Antibiotics were quantified using ultra-high performance liquid chromatography (UHPLC, Vanquish, Thermo Scientific, USA) in tandem with a triple quadrupole mass spectrometer (MS, TSQ Endura, Thermo Scientific, USA) equipped with an electrospray ionization (ESI) source. Detailed parameters for UHPLC and MS are given in Tables S2 and S3.

The water quality and characteristics of sediments were also determined, and the results are shown in Tables S4 and S5.

### Quality assurance and quality control

For each sampling site, two parallel samples were conducted. In each sampling campaign, at least one procedural blank was prepared using deionized water. Internal standard method was used to quantify the concentrations of antibiotics. Seven isotopically labelled internal standards, almost one isotopic internal standard for each class of antibiotics, were used (Table S2). Identification of target antibiotics was based on matching the retention time and quantitative and qualitative ions with their corresponding standards. A control sample (standard at 50 µg/L) and two solvent blanks (methanol) were inserted in the sequence every 20 injections to assess the potential contamination and stability of instrument. The limit of detection (LOD) was defined as the concentration that yielded a signal-to-noise ratio of 3:1 and considering the concentration factor. The LOD varied from 0.001 to 0.35 ng/L for water sample and from 0.001 to 0.14 ng/L for sediment samples^18^. All the procedural and solvent blank samples showed antibiotic concentrations below LOD, indicating no pollution during sample pretreatment and analysis. Relative standard deviation (RSD) of check standards and duplicate injections were much lower than 10%, indicating good repeatability of analysis.

### Data collection of antibiotic concentrations from other urban rivers in China

To compare the antibiotic concentrations of the present study with those from other urban rivers in China, a literature review was performed using the method in our previous study^5^ up to February 14, 2020. Totally, 84 relevant papers (48 papers in English and 36 papers in Chinese) were selected. According to the economic development and geographical location of different provinces, China is divided into 5 regions in this study: Bohai Rim, Yangtze River Delta, Southeast Coastal Economic Zone, Northeast China, and Central and western China (Figure S2).

Twelve antibiotics (sulfadiazine (SDZ), sulfamethazine (SMZ), SMX, oxytetracycline (OTC), TC, ofloxacin (OFL), norfloxacin (NOR), CIP, enrofloxacin (ENR), erythromycin (ETM), roxithromycin (RTM), and clarithromycin (CTM)), which were detected in all the 5 regions of China, were selected to compare the regional differences of antibiotic concentrations in urban river water of China. The antibiotic concentrations in sediments were not compared because fewer data are available.

### Environmental risk assessment

The possible risk to antimicrobial resistance development, risk quotient (RQ_AMR_), was calculated based on the measured environmental concentration (MEC) in water and minimal predicted no effect concentration for antimicrobial resistance development (PNEC_AMR_) as described in Eq. (1).

1 $${\text{RQ}}_{{{\text{AMR}}}} = {\text{MEC}}/{\text{PNEC}}_{{{\text{AMR}}}}$$

where PNEC_AMR_ values were obtained from previous studies^19,20^ and are shown in Table S6. The risk is divided into two levels: low risk (RQ_AMR_ < 1) and possible risk (RQ_AMR_ ≥ 1).

The ecological risks of antibiotics were predicted using risk quotient (RQ), and RQ for each antibiotic was calculated using the method described in Text S1^16,17^. The toxicity data of antibiotics to fish, daphnid, and green algae, obtained from ECOSAR (v2.0, USEPA), are given in Table S7. The toxicity data to bacteria were acquired from previous literature (Table S11).

### Statistical analysis

Two-sample *t*-test was employed to evaluate the significant differences in antibiotics between two seasons. Principle component analysis (PCA) was performed by OriginPro 2018 (Academic) to investigate the compositional differences in antibiotics between different samples. Heatmaps were plotted by using OriginPro 2018 (Academic). Sankey diagrams, the analysis of similarity (ANOSIM), and redundancy analysis (RDA) were conducted using R studio (Version 1.1.447). Sampling sites and land use maps were drawn in ArcGIS 10.2.

## Results and discussion

### Antibiotic concentrations in the Huangshui River

**Figure 2:**
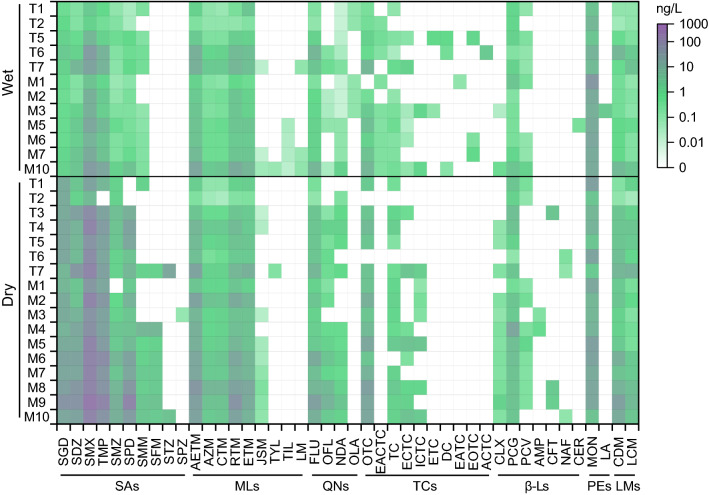
Antibiotic concentrations in water samples from the Huangshui River.

A total of 48 antibiotics were detected in the Huangshui River over the two sampling campaigns (Figure S3). Among them, 44 and 35 antibiotics were detected in water and sediments, respectively. The concentrations of individual antibiotics varied from undetected (ND) to 552 ng/L in water (Fig. 2) and from ND to 164 ng/g in sediments (Figure S4), which are lower than those in water (ND–2702 ng/L) and sediments (ND–449 ng/g) in urban black-odor rivers in Guangzhou, South China^13^. Among antibiotic families, SMX (1.17–552 ng/L), sulfaguanidine (SGD) (0.59–56.6 ng/L), and SDZ (0.22–58.9 ng/L) in SAs, anhydroerythromycin (AETM) (0.41–46.9 ng/L), azithromycin (AZM) (0.08–7.67 ng/L), CTM (0.06–7.75 ng/L), ETM (0.30–16.6 ng/L), and RTM (0.12–48.7 ng/L) in MLs, flumequine (FLU) (0.11–26.1 ng/L) in QNs, penicillin G (PCG) (0.16–12.1 ng/L) in β-Ls, monensin (MON) (2.14–43.2 ng/L) in PEs, and clindamycin (CDM) (0.08–30.4 ng/L) and lincomycin (LCM) (0.07–8.62 ng/L) in LMs were detected in all water samples (detection frequencies 100%) (Figure S3). In sediments, AETM (0.03–1.68 ng/g), AZM (0.03–0.87 ng/g), CTM (0.02–0.86 ng/g), and CDM (< LOQ–1.25 ng/g) showed detection frequencies of 100% (Figure S4 and S5).

**Figure 3:**
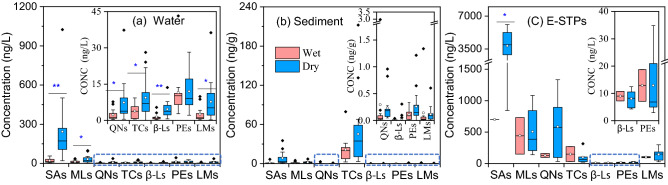
Comparison of antibiotic concentrations in wet and dry seasons (Significant difference: *p < 0.05, **p < 0.01).

In line with previous studies^16,21^, much higher concentrations of different classes of antibiotics in water were observed in dry season than those in wet season (*p* < 0.05) except for PEs (Fig. 3a). Seasonal variation in antibiotic concentrations mainly depends on water flow and consumption patterns^22^. More precipitation in wet season (from June to September), accounting for 70% of the annual precipitation, was recorded in the studied area^15^. Hence, the dilution effect in wet season resulted in lower antibiotic concentrations in river water. On the other aspect, greater usage of antibiotics in cold (dry) season may also induce their high mass load in surface water^23,24^. In addition, biodegradation, hydrolysis, and photodegradation are more active in warm (wet) season^25^. In contrast, seasonal differences in the concentrations of different classes of antibiotics were insignificant for sediment samples (Fig. 3b). This is probably because the antibiotics in sediments reflect the accumulative effect of pollutants.

Comparing different classes of investigated antibiotics, SAs, with total concentrations of 21.3–699 ng/L and 19.5–1025 ng/L in wet and dry seasons, respectively, were predominant in the Huangshui River water (Fig. 3a). High SAs concentrations were often observed in natural rivers^5^, because of their persistence to degradation as well as low potential to be adsorbed by sediments resulted from their low log*K*_ow_ values (Table S1). However, TCs, with total concentrations of 2.53–79.5 ng/g and 3.14–212 ng/g in wet and dry seasons, respectively, dominated in sediments (Fig. 3b). This is ascribed to their direct surface complexation with metals and different functional groups of sediment organic matters^26^.

### Antibiotic concentrations in E-STPs

A total of 44 antibiotics were detected in E-STPs (Figure S2a). Among them, 30 antibiotics were also detected in both water and sediments of the river. The levels of individual antibiotics ranged from ND to 3821 ng/L in E-STPs (Figure S6), indicating the incomplete removal of antibiotics by various treatment processes used in STPs^10,22^. SMX (420–3821 ng/L), trimethoprim (TMP, 94.7–2340 ng/L), and OFL (4.13–1103 ng/L) were dominant in E-STPs (Figure S6). High concentrations of individual antibiotics up to several μg/L were also detected in other E-STPs in the world, e.g. 0.011–8.3, 0.001–1.8, and 0.024–6.8 μg/L for SMX, TMP, and OFL, respectively^9^. SAs, QNs, and MLs were also frequently detected in STPs worldwide^27^.

Similar to the river water (Fig. 3a), the total concentrations of SAs, varying from 700 to 6103 ng/L, accounted for the majority of the total concentrations of antibiotics in E-STPs (Fig. 3c). Moreover, a significant seasonal difference (*p* < 0.05) was only observed for the total concentrations of SAs (Fig. 3c), revealing the heavy mass load of SAs in cold season due to the prevalence of respiratory diseases^22,28^. The insignificant seasonal differences in concentrations of other classes of antibiotics may indicate the relatively high and stable removal of these antibiotics in STPs.

### Comparison of antibiotic compositions in river water, sediments, and E-STPs

**Figure 4:**
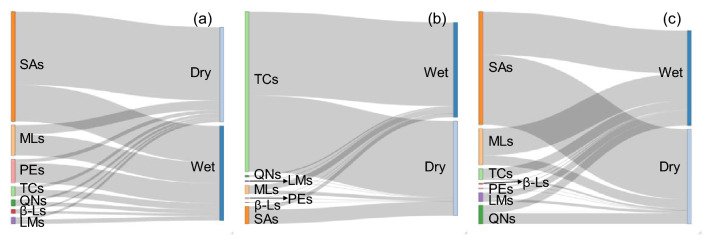
Sankey diagrams of antibiotics in water (**a**), sediments (**b**), and E-STPs (**c**).

Sankey diagrams (Fig. 4) displayed the average compositions of different classes of antibiotics in different types of samples. It was shown that SAs dominated in river water (38.7–77.1%, Fig. 4a) and E-STPs (45.4–73.9%, Fig. 4c) in both seasons. MLs were the second dominant antibiotics in river water, accounting for 22.0% and 10.0% of the total concentration in wet season and dry season, respectively (Fig. 4a). In E-STPs, MLs and QNs accounted for 36.9% and 21.1%, respectively, of the total concentration in wet season and dry season (Fig. 4c). In contrast, TCs were the predominant antibiotics in sediments, accounting for 88.3% in wet season and 80.4% in dry season of the total antibiotic concentrations, respectively. This is mainly ascribed to the adsorption of TCs by sediments^29^. The preferable adsorption of some antibiotics leads to their redistribution between water and sediments^30^. It has been reported that TCs are highly adsorbed to clay minerals, soils, and sediments^31–33^. Moreover, the presence of metals, e.g. Cu(II) and Cd(II), can facilitate the adsorption of TCs by sediments due to their complexation with metal ions^29,34^.

The very low concentrations of β-Ls in all the three types of samples were attributed to their high susceptibility to chemical or enzymatic hydrolysis in STPs^27,35^. It has been reported that chemical hydrolysis and/or transformations of β-Ls antibiotics can take place under acidic/alkaline conditions or catalyzed by metals^37^. The lower proportions of QNs in river water and sediments in comparison to that in E-STPs were attributed to their susceptibility to photolysis^38^ as well as their limited use^3^. The lower contributions of QNs to the total antibiotics were widely found in water and sediments from various surface waters^5,17^.

**Figure 5:**
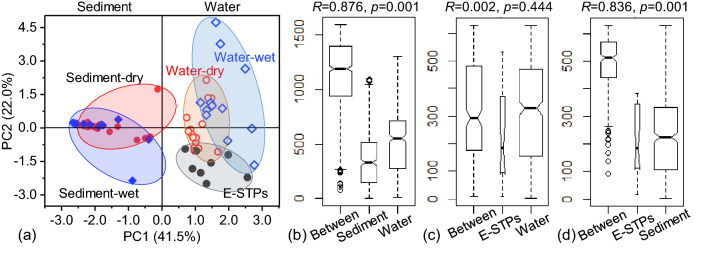
PCA plot of antibiotic composition (**a**) and the ranks of the dissimilarities in antibiotic compositions between water, sediments, and E-STPs (**b**–**d**).

To further investigate the compositional differences in antibiotics between different samples, PCA was performed using the normalized concentrations for each class of antibiotics by total antibiotic concentrations. Two PCs were identified, explaining 41.5% and 22.0% of the total variances for PC1 and PC2, respectively (Fig. 5a). Seasonal differences of antibiotic compositions in water or sediments were not observed. However, PC1 revealed that the antibiotic compositions in water were different from those in sediments. This was confirmed by ANOSIM statistic test of pairwise Bray–Curtis dissimilarities (Fig. 5b), which displayed a significant difference in antibiotic compositions between water and sediment (*R* = 0.876, *p* = 0.001).

Moreover, the sample clusters of water and E-STPs were overlapped but were well separated from those of sediments (Fig. 5a), suggesting that the antibiotic compositions in E-STPs were close to those in water samples but different from those in sediments. This was also confirmed by the ANOSIM statistic test, which showed an insignificant difference between water and E-STPs (*R* = 0.002, *p* = 0.444) but a significant difference between E-STPs and sediments (*R* = 0.836, *p* = 0.001) (Fig. 5c,d). The similarities of antibiotic compositions between E-STPs and river water indicate the impact of E-STPs on its receiving water.

### Spatial distribution of antibiotics

**Figure 6:**
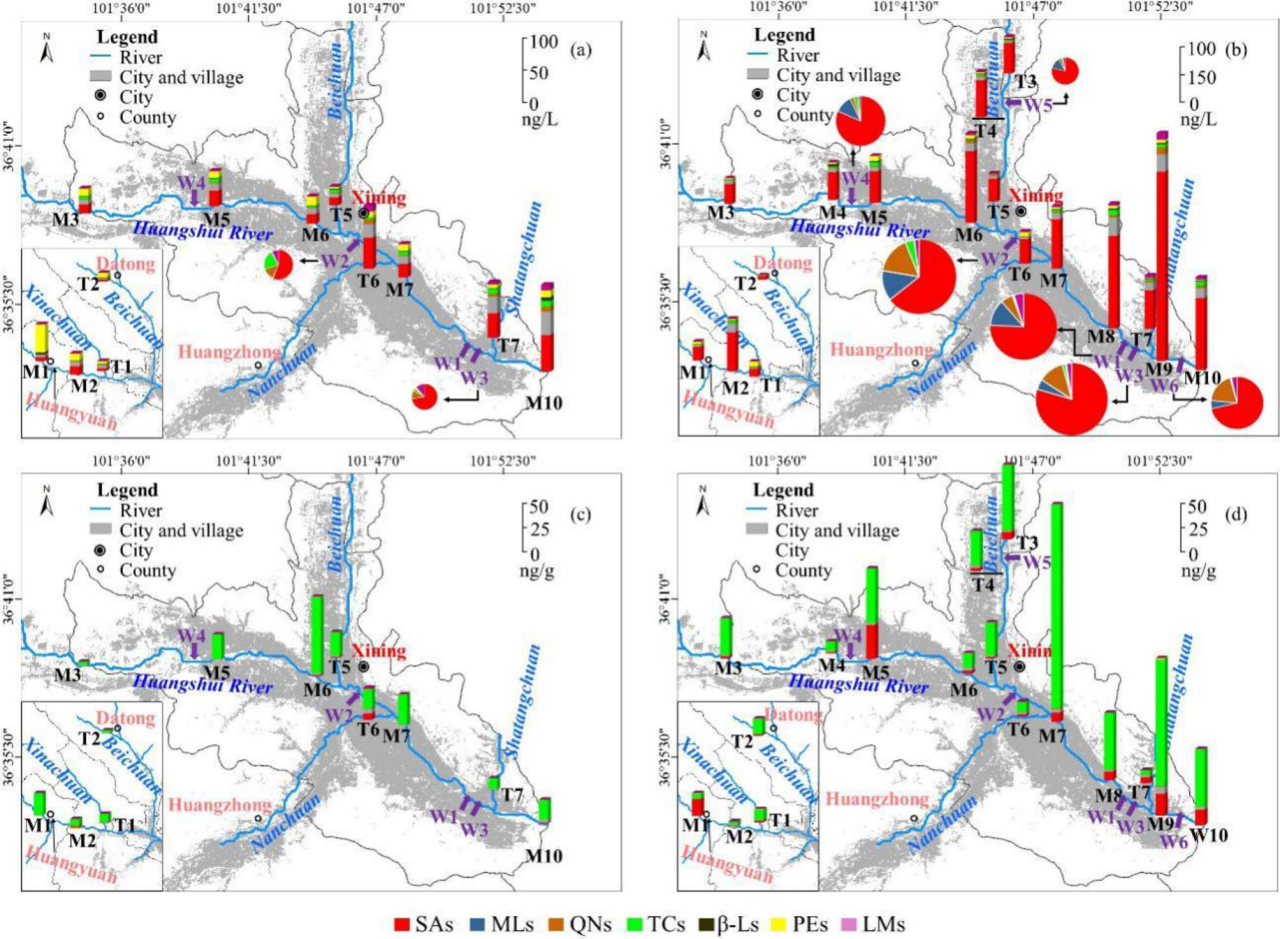
Spatial distribution of antibiotics in the Huangshui River and E-STPs (a and b, water in wet and dry season; c and d, sediments in wet and dry season). Maps generated using ArcGIS 10.2‒ https://developers.arcgis.com; Image from https://data.ess.tsinghua.edu.cn.

A great spatial variation in the total concentrations of antibiotics were observed in river water (13.8–1234 ng/L) and sediments (3.19–227 ng/g) (Fig. 6). Moreover, higher concentrations of antibiotics occurred in urban area (M4–M10 and T3–T7) than those in suburban area (M1–M3 and T1–T2). In urban areas, the continuous discharge of E-STPs resulted in the high levels of antibiotics in the receiving water^21,28^. Indeed, elevated concentrations of antibiotics were observed downstream of the STPs, e.g. the elevated antibiotic concentrations at M5 downstream of W4, and the extremely high antibiotic concentrations in water and sediments at M9 and M10 after W1 and W3. Furthermore, the large mass load of antibiotics from STPs into the river in dry season resulted in extremely high antibiotic concentrations in river water (Fig. 6b). In addition, a small amount of untreated wastewater from households might be the major source of antibiotics at M8 (Fig. 6b).

On the other hand, the runoff from manured farmland may also contribute to the antibiotic pollution in suburban area^28^. This may explain the higher proportion of PEs in wet season (Fig. 6a), especially in rural area of the Huangshui River basin (M1–M3 and T1–T2), because PEs are used as feed additives for poultry and livestock and growth promotors for ruminants^35,39^. High concentrations of PEs (MON and salinomycin (SAL)) were found in water collectors adjacent to intensive-husbandry facilities^40^. As shown in the land use pattern of the studied area (Figure S7), M1–M3 and T1–T2 are located in the rural area with cropland around. The influence of croplands applied manure on the contaminants in the aquatic environments has also been reported in previous studies^28,40^.

Comparing different STPs (Fig. 6b), the antibiotic concentrations in effluents of W1–W3 were much higher than those in effluents of W4–W6. This may be related to the treatment processes used in STPs (Table S8). In general, anaerobic/anoxic/oxic (A/A/O) and oxidation ditch (OD) in STPs achieved relatively high removal of micropollutants among various secondary treatment processes^41^. Moreover, moving bed biofilm reactor (MBBR), following A/A/O, can further remove some micropollutants^42^. However, the increased removal of antibiotics by A/AO-MBBR was not observed in W3 compared with W1 (A/AO + JS-BC) and W2 (OD). Obviously, the STPs with a tertiary treatment process of UV disinfection (W5 and W6) or chemical phosphorus removal (W4) (Table S8) increased the removal of antibiotics compared with those STPs with secondary treatment processes (W1–W3)^41^.

RDA was conducted to investigate the relationships between antibiotic concentrations and environmental factors. It was demonstrated that the concentrations for most of the investigated antibiotics correlated with nitrogen (total nitrogen (TN), nitrate nitrogen (NO_3_^–^N), nitrite nitrogen (NO_2_^–^N), and ammonium nitrogen (NH_4_^+^-N)) in river water (Figure S8a and b), indicating their same pollution source. For sediments, most of the antibiotics showed positive relationships with nitrogen, total phosphorus (TP), and sediment organic carbon (SOC) (Figure S8 c and d). It is widely accepted that SOC is one of the most important factors to determine the antibiotic concentrations in sediments^16,43^.

### Comparison with other urban rivers in China

**Figure 7:**
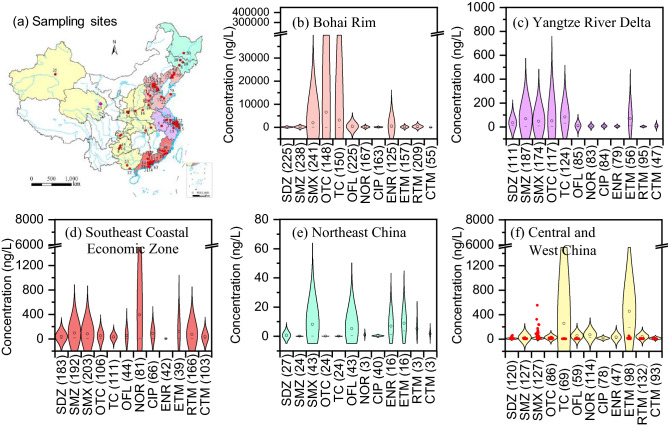
Comparison of antibiotic concentrations in urban river water from different regions of China (The numbers in brackets are data number; The colors in the map (**a**), which represent different regions, correspond to the colors in the violin plot (**b**–**f**); The white color in the map (**a**) means no data available in this province; Red dots in violin plot (**f**) are the data from Huangshui River). Map created using ArcGIS 10.2‒ https://developers.arcgis.com; Image from https://www.resdc.cn/Default.aspx.

It is difficult to compare the total concentrations and compositions of antibiotics among different rivers, because of the inconsistency in the target antibiotics investigated in various studies. Therefore, only 12 antibiotics, detected in all the 5 regions of China, were selected for comparison (Table S9). However, it is noted that, among these antibiotics detected in all the regions, NOR, CIP, and ENR, belonging to QNs, were not detected in the Huangshui River water (Fig. 7f). CIP was not detected in river sediments and E-STPs either, which may be attributed to their lower usage in this area. Whereas, NOR were detected in both river sediments and E-STPs, and ENR were only detected in E-STP. Thus, it was speculated that the disappearance of NOR and ENR in river water may reflect the influence of the special climate conditions in plateau areas. The strong solar radiation and long sunshine hours in this plateau area^15^ induced favored photolysis of QNs^38^. Consequently, NOR and ENR occurred in E-STPs and/or sediments but were not detected in the river water due to their photolysis induced by strong solar radiation.

As shown in Fig. 7 and Table S9, the concentration of antibiotics in urban river water varied significantly, up to 8 orders of magnitude. Among different regions of China, high concentrations of antibiotics occurred in urban rivers from Bohai Rim (ND–387,000 ng/L, mean 9.80–3735 ng/L) (Fig. 7b and Table S9). It is an economically developed region (gross domestic product (GDP) of 47,772–140,211 RMB per capita) with high population density of 294–1312 person/km^2^ and COD discharge of 1.71–7.78 t/km^2^a (Table S10), which result in high emission of antibiotics^3^. In contrast, the urban rivers in Northeast China demonstrated relatively low antibiotic concentrations (ND–73.1 ng/L) than those in other regions (*p* < 0.05), which may partially be ascribed to the limited data in this region (Fig. 7e). The concentrations of most antibiotics in the Huangshui River were in the ranges of those in urban rivers from central and western China, except for SMX and SDZ with relatively large concentration ranges (Fig. 7f). As shown in Table S9, the levels of antibiotics in the Huangshui River were comparable to the levels in urban rivers in Japan (ND–233 ng/L) and USA (ND–576 ng/L), but lower than those in urban rivers in Vietnam (< LOQ–48,517 ng/L), Brazil (ND–1800 ng/L) and Australia (ND–2000 ng/L).

Figure 7 also displayed obvious differences in the antibiotic compositions for various regions of China. Comparing different kinds of antibiotics, notably, the urban rivers from Bohai Rim showed extremely high concentrations of SMX (ND–145,290 ng/L), OTC (ND–361,107 ng/L), and TC (ND–387,000 ng/L), and their mean concentrations were up to several thousands ng/L (2049 ng/L for SMX, 3735 ng/L for OTC, and 3216 ng/L for TC). The urban rivers in the Yangtze River demonstrated high concentration of SMZ (ND–930 ng/L), SMX (0.10–765 ng/L), OTC (ND–2260 ng/L) and TC (ND–1000 ng/L). The concentrations of all the 12 antibiotics in urban rivers from Southeast Coastal Economic Zone were relatively high (ND–2479 ng/L) except for NOR with high and wide concentrations range (ND–6620 ng/L) and ENR with low and narrow concentration range (ND–2260 ng/L). The urban rivers in central and western China demonstrated extremely high and wide range of TC (ND–6800 ng/L) and ETM (ND–2910 ng/L). The great regional differences in the concentrations and compositions of antibiotics depend mainly on usage patterns, population density, weather conditions, environmental persistence of antibiotics, and elimination efficiency of STPs^23,24,27^.

### Ecological risk assessment

**Figure 8:**
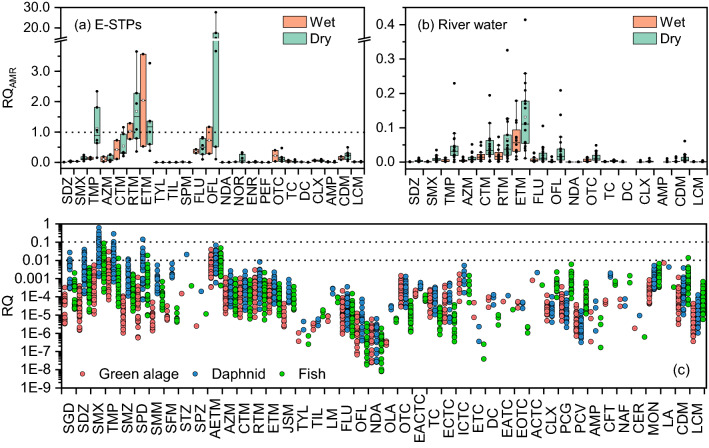
The possible risks to antimicrobial resistance development (RQ_AMR_) for antibiotics in E-STPs (**a**) and river water (**b**) and potential ecological risks (RQ) to aquatic organism for antibiotics in river water (**c**).

The presence of antibiotics in E-STPs and river water may promote the development of antibiotic resistance bacteria and ARGs^7^. The concentrations of many antibiotics detected in E-STPs (Figure S1) were close to or even exceeding PNEC_AMR_ (Table S7). OFL, ETM, RTM, CTM, and TMP showed RQ_AMR_ values higher than 1 (Fig. 8a), suggesting that these antibiotics may have possible risks to the evolution of antibiotic resistance. Therefore, the occurrence of antibiotics in E-STPs should be given special attention, because the E-STPs is used for irrigation of plants and grass in urban areas, which may increase the possible risk to public health. Tran et al. (2019)^44^ also found that ETM, CTM, TMP, and OFL showed a possible risk for antibiotic resistance evolution in urban canals in Hanoi, Vietnam.

In contrast, all the individual antibiotic in river water (Fig. 2) showed concentrations lower than their corresponding PNEC_AMR_ values (Table S7), and the RQ_AMR_ values were all below 0.45 (Fig. 8b). This indicates low risk for antibiotic resistance development in the Huangshui River. Similar to those in E-STPs, ETM, RTM, and CTM in MLs, TMP in SAs, and FLU and OFL in QNs showed relatively higher risks for antibiotic resistance development compared with other antibiotics. On the other hand, as we known, the bacteria in STPs as well as in the river are exposed to the mixtures of antibiotics rather than a single chemical. This may make the bacteria more sensitive^19^. Particularly, not only antibiotics but also other contaminants, e.g. metals, antiepileptic drugs, and non-antibiotic antimicrobial, can induce antibiotic resistance^45,46^. Moreover, multiple antibiotic resistance frequently occurs in the environment^47^. Therefore, it is worthwhile to further investigate the occurrence and distribution of ARGs in the Huangshui River.

Apart from resistance selection, the discharge of effluents containing antibiotics into the receiving water bodies can pose a serious threat to aquatic organisms. As shown in Fig. 8c, most of the antibiotics displayed insignificant risks to green algae, daphnid, and fish. Among them, TMP and AETM had low to medium risks to green algae in very few sampling sites; SMX, TMP, and SPD in SAs could induce medium risks to daphnid with RQ values higher than 0.1 but lower than 1; SMX, TMP, SPD, AETM, and CDM posed low risks to fish in some sampling sites. The RQ values of antibiotics to bacteria were calculated based on the toxicity data obtained from toxicological experiments (Table S11), which are shown in Figure S9. Most antibiotics posed insignificant risks to bacteria. Among them, OFL, OLA, OTC, TC, ETC, and DC displayed low to medium risks to bacteria. Although high risks (RQ > 1) were not found for antibiotics in the Huangshui River, a lack of information on the synergistic impact of these chemicals, particularly at low concentration over longer exposure times is still a matter of great concern^19,27^. Another concern is the discharge of the metabolites of antibiotics, which may lead to greater ecological or human health risks than their parent compounds^48^.

## Conclusions

This study investigated 83 antibiotics in water and sediments from the Huangshui River, and E-STPs in Xining, Qinghai, in the northeastern Tibetan plateau. The concentrations of individual antibiotics varied from ND to 552 ng/L in river water, from ND to 164 ng/g in river sediments, and from ND to 3821 ng/L in E-STPs. Higher concentrations in dry season than wet season were observed due to less precipitation and greater usage of antibiotics in dry (cold) season. SAs predominated in river water and E-STPs, however, TCs predominated in river sediments. Higher concentrations of antibiotics occurred in urban area than those in suburban area due to the discharge of E-STPs in urban area. The STPs with a tertiary treatment process of UV disinfection or chemical phosphorus removal showed lower antibiotic concentrations in their effluents than those with secondary treatment processes. OFL, ETM, RTM, CTM, and TMP in E-STPs showed RQ_AMR_ values higher than 1, suggesting their possible potential risk to antibiotic resistance evolution. TMP, AETM, SMX, SPD, and CDM in river water could cause low to medium risks to green algae, daphnid, and/or fish, while OFL, OLA, OTC, TC, ETC, and DC could induce low to medium risks to bacteria.

## Supplementary information

Supplementary Information.

## References

[1] Cabello FC (2006). Heavy use of prophylactic antibiotics in aquaculture: a growing problem for human and animal health and for the environment. Environ. Microbiol..

[2] Sarmah AK, Meyer MT, Boxall AB (2006). A global perspective on the use, sales, exposure pathways, occurrence, fate and effects of veterinary antibiotics (VAs) in the environment. Chemosphere.

[3] Zhang Q, Ying G, Pan C, Liu Y, Zhao J (2015). Comprehensive evaluation of antibiotics emission and fate in the river basins of China: source analysis, multimedia modeling, and linkage to bacterial resistance. Environ. Sci. Technol..

[4] Yin X (2013). A systematic review of antibiotic utilization in China. J. Antimicrob. Chemother..

[5] Li S (2018). A duodecennial national synthesis of antibiotics in China's major rivers and seas (2005–2016). Sci. Total Environ..

[6] Wilkinson, J. & Boxall, A. The first global study of pharmaceutical contamination in riverine environments. in SETAC Europe 29th Annual Meeting, Helsinki, May 28 (2019).

[7] Danner MC, Robertson A, Behrends V, Reiss J (2019). Antibiotic pollution in surface fresh waters: occurrence and effects. Sci. Total Environ..

[8] Levin-Reisman I, Ronin I, Gefen O, Braniss I, Shoresh N, Balaban NQ (2017). Antibiotic tolerance facilitates the evolution of resistance. Science.

[9] Oberoi AS, Jia Y, Zhang H, Khanal SK, Lu H (2019). Insights into the fate and removal of antibiotics in engineered biological treatment systems: a critical review. Environ. Sci. Technol..

[10] Rodriguez-Mozaz S (2015). Occurrence of antibiotics and antibiotic resistance genes in hospital and urban wastewaters and their impact on the receiving river. Water Res..

[11] Michael I (2013). Urban wastewater treatment plants as hotspots for the release of antibiotics in the environment: a review. Water Res..

[12] Xu J (2015). Occurrence of antibiotics and antibiotic resistance genes in a sewage treatment plant and its effluent-receiving river. Chemosphere.

[13] Huang Y (2019). Occurrence and distribution of antibiotics and antibiotic resistant genes in water and sediments of urban rivers with black-odor water in Guangzhou South China. Sci. Total Environ..

[14] Jia J (2018). Occurrence and distribution of antibiotics and antibiotic resistance genes in Ba River China. Sci. Total Environ..

[15] Mao X, Wei X, Jin X, Tao Y, Zhang Z, Wang W (2019). Monitoring urban wetlands restoration in Qinghai Plateau: integrated performance from ecological characters, ecological processes to ecosystem services. Ecol. Indic..

[16] Li S (2018). Antibiotics in water and sediments of rivers and coastal area of Zhuhai City, Pearl River estuary, south China. Sci. Total Environ..

[17] Li S (2019). Antibiotics in water and sediments of Danjiangkou Reservoir, China: spatiotemporal distribution and indicator screening. Environ. Pollut..

[18] Li S (2020). Enrichment of antibiotics in an inland lake water. Environ. Res..

[19] Bengtsson-Palme J, Larsson DGJ (2016). Concentrations of antibiotics predicted to select for resistant bacteria: proposed limits for environmental regulation. Environ. Int..

[20] Kümmerer K, Henninger A (2003). Promoting resistance by the emission of antibiotics from hospitals and households into effluent. Clin. Microbiol. Infect..

[21] Lei K (2019). Spatial and seasonal variations of antibiotics in river waters in the Haihe River Catchment in China and ecotoxicological risk assessment. Environ. Int..

[22] Patel M, Kumar R, Kishor K, Mlsna T, Pittman CU, Mohan D (2019). Pharmaceuticals of emerging concern in aquatic systems: chemistry, occurrence, effects, and removal methods. Chem. Rev..

[23] Petrie B, Barden R, Kasprzyk-Hordern B (2015). A review on emerging contaminants in wastewaters and the environment: current knowledge, understudied areas and recommendations for future monitoring. Water Res..

[24] Vatovec C, Phillips P, Van Wagoner, E, Scott TM, Furlong E (2016). Investigating dynamic sources of pharmaceuticals: demographic and seasonal use are more important than down-the-drain disposal in wastewater effluent in a University City setting. Sci. Total Environ..

[25] Lindholm-Lehto PC, Ahkola HS, Knuutinen JS, Herve SH (2016). Widespread occurrence and seasonal variation of pharmaceuticals in surface waters and municipal wastewater treatment plants in central Finland. Environ. Sci. Pollut. Res..

[26] Huang Y (2020). Impact of sediment characteristics on adsorption behavior of typical antibiotics in Lake Taihu China. Sci. Total Environ..

[27] Tran NH, Reinhard M, Gin KYH (2018). Occurrence and fate of emerging contaminants in municipal wastewater treatment plants from different geographical regions-a review. Water Res..

[28] Su D, Ben WW, Strobel BW, Qiang Z (2020). Occurrence, source estimation and risk assessment of pharmaceuticals in the Chaobai River characterized by adjacent land use. Sci. Total Environ..

[29] Zhang Z, Sun K, Gao B, Zhang G, Liu X, Zhao Y (2011). Adsorption of tetracycline on soil and sediment: effects of pH and the presence of Cu(II). J. Hazard. Mater..

[30] Perelo LW (2010). Review: in situ and bioremediation of organic pollutants in aquatic sediments. J. Hazard. Mater..

[31] Gu C, Karthikeyan KG (2008). Sorption of the antibiotic tetracycline to humic-mineral complexes. J. Environ. Qual..

[32] Li Z, Chang P, Jean JS, Jiang W, Wang C (2010). Interaction between tetracycline and smectite in aqueous solution. J. Colloid Interface Sci..

[33] ter Laak TL, Gebbink WA, Tolls J (2006). Estimation of soil sorption coefficients of veterinary pharmaceuticals from soil properties. Environ. Toxicol. Chem..

[34] Chen Z, Li G, Sun L, Li Y (2016). Tetracyclines sorption in the presence of cadmium on river sediments: The effects of sorption mechanism and complex properties. Water Air Soil Pollut..

[35] Cha J, Yang S, Carlson KH (2015). Occurrence of β-lactam and polyether ionophore antibiotics in surface water, urban wastewater, and sediment. Geosystem Eng..

[36] Chen J, Sun P, Zhou X, Zhang Y, Huang C (2015). Cu(II)-catalyzed transformation of benzylpenicillin revisited: the overlooked oxidation. Environ. Sci. Technol..

[37] Chen J, Sun P, Zhang Y, Huang C (2016). Multiple roles of Cu(II) in catalyzing hydrolysis and oxidation of beta-lactam antibiotics. Environ. Sci. Technol..

[38] Avisar D, Lester Y, Mamane H (2010). pH induced polychromatic UV treatment for the removal of a mixture of SMX, OTC, and CIP from water. J. Hazard. Mater..

[39] Westley, J.W. Polyether antibiotics. (ed. Westley, J.) Ch. 6. (New York: Marcel Dekker, 1982).

[40] Alonso LL, Demetrio PM, Capparelli AL, Marino DJG (2019). Behavior of ionophore antibiotics in aquatic environments in Argentina: the distribution on different scales in water courses and the role of wetlands in depuration. Environ. Int..

[41] Ben W, Zhu B, Yuan X, Zhang Y, Yang M, Qiang Z (2018). Occurrence, removal and risk of organic micropollutants in wastewater treatment plants across China: comparison of wastewater treatment processes. Water Res..

[42] Falås P, Longree P, Jansen JLC, Siegrist H, Hollender J, Joss A (2013). Micropollutant removal by attached and suspended growth in a hybrid biofilm activated sludge process. Water Res..

[43] Liu X (2016). Levels, distributions and sources of veterinary antibiotics in the sediments of the Bohai Sea in China and surrounding estuaries. Mar. Pollut. Bull..

[44] Tran NH (2019). Occurrence and risk assessment of multiple classes of antibiotics in urban canals and lakes in Hanoi Vietnam. Sci. Total Environ..

[45] Lu J (2018). Triclosan at environmentally relevant concentrations promotes horizontal transfer of multidrug resistance genes within and across bacterial genera. Environ. Int..

[46] Wang Y (2019). Antiepileptic drug carbamazepine promotes horizontal transfer of plasmid-borne multi-antibiotic resistance genes within and across bacterial genera. ISME J..

[47] Qiao M, Ying G, Singer AC, Zhu Y (2018). Review of antibiotic resistance in China and its environment. Environ. Int..

[48] Haddad T, Baginska E, Kuemmerer K (2015). Transformation products of antibiotic and cytostatic drugs in the aquatic cycle that result from effluent treatment and abiotic/biotic reactions in the environment: an increasing challenge calling for higher emphasis on measures at the beginning of the pipe. Water Res..

