# Supplementary material for: Occurrence and risks of antibiotics in an urban river in northeastern Tibetan Plateau
Source: Sci Rep. 2020 Nov 18;10:20054. doi: 10.1038/s41598-020-77152-5 (PMC7675971; doi:10.1038/s41598-020-77152-5)
Supplement: Supplementary file 1 — Supplementary Information. [file 41598_2020_77152_MOESM1_ESM.pdf]

Supporting Information

Occurrence and risks of antibiotics in an urban river in northeastern Tibetan plateau

Yuzhu Kuang[1], Xiaoyu Guo[1], Jingrun Hu[2], Si Li[3], Ruijie Zhang[2], Qiang Gao[4], Xi Yang[4], Qian Chen[4], Weiling Sun[2,4*]

[1] *College of Resources Environment and Tourism, Capital Normal University, Beijing 100048, China*

[2] *College of Environmental Sciences and Engineering, Peking University, State Environmental Protection Key Laboratory of All Materials Flux in River Ecosystems, The Key Laboratory of Water and Sediment Sciences, Ministry of Education, International Joint Laboratory for Regional Pollution Control, Ministry of Education, Beijing 100871, China*

[3] *Beijing Key Laboratory of Farmland Soil Pollution Prevention and Remediation, College of Resources and Environmental Sciences, China Agricultural University, Beijing 100193, China*

[4] *State Key Laboratory of Plateau Ecology and Agriculture, Qinghai University, Xining 810016, China*

[*] Corresponding author. E-mail: wlsun@pku.edu.cn (W.L. Sun)

Text S1 Ecological risk assessment

Ecological risk of antibiotics was evaluated according to the European technical guidance document on risk assessment[1]. RQ was calculated through the measured environmental concentration (MEC) divided by the predicted no-effect concentration (PNEC).

$$RQ = MEC/PNEC \quad (S1)$$

PNEC was calculated by dividing acute toxicity data or chronic toxicity data with an assessment factor (AF).

$$PNEC = EC_{50}(LC_{50})/AF \text{ or } PNEC = chv/AF \quad (S2)$$

where $EC_{50}(LC_{50})$ represent the median effective or lethal concentrations, chv represents chronic toxicity. The values of AF were 1000 and 100 for acute and chronic toxicity, respectively[1-2].

Chronic toxicity data were preferred for assessing the risk, and the chronic values to fish, daphnid, and green algae in water were predicted by ECOSAR database (v2.0, USEPA) in this study (Table S7). The toxicity data to bacteria were acquired from previous literature (Table S8). The levels of risk could be divided into four categories, i.e., insignificant risk ($RQ < 0.01$), low risk ($0.01 < RQ < 0.1$), medium risk ($0.1 < RQ < 1$), and high risk ($RQ > 1$)[3].

Table S1 The properties of the target antibiotics.

Class	Antibiotic	Abbreviation	Molecular formula	CAS No.	Molecular weight (g/mol)	Log Kow [a]	pKa
SAs	Sulfabenzamide	SBZ	$C_{13}H_{12}N_2O_3S$	127-71-9	276.3	1.30	2.09, 4.32 [e]
	Sulfachloropyridazine	SCP	$C_{10}H_9ClN_4O_2S$	80-32-0	284.7	0.31	1.87, 5.45 [f]
	Sulfadiazine	SDZ	$C_{10}H_{10}N_4O_2S$	68-35-9	250.3	-0.09	2.00, 6.48 [b]
	Sulfadimethoxine	SDM	$C_{12}H_{14}N_4O_4S$	122-11-2	310.3	1.63	1.87, 5.86 [g]
	Sulfadoxine	SDX	$C_{12}H_{14}N_4O_4S$	2447-57-6	310.3	0.70	1.52, 6.01 [h]
	Sulfamerazine	SMR	$C_{11}H_{12}N_4O_2S$	127-79-7	264.3	0.14	2.06, 6.90 [b]
	Sulfameter	SFM	$C_{11}H_{12}N_4O_3S$	651-06-9	280.3	0.41	1.87, 6.50 [g]
	Sulfamethazine	SMZ	$C_{12}H_{14}N_4O_2S$	57-68-1	278.3	0.89	2.65, 7.65 [b]
	Sulfamethizole	SMTZ	$C_9H_{10}N_4O_2S_2$	144-82-1	270.3	0.54	1.86, 5.29 [f]
	Sulfamethoxazole	SMX	$C_{10}H_{11}N_3O_3S$	723-46-6	253.3	0.89	1.85, 5.60 [f]
	Sulfamethoxypyridazine	SMP	$C_{11}H_{12}N_4O_3S$	80-35-3	280.3	0.32	2.09, 6.95 [g]
	Sulfamonomethoxine	SMM	$C_{11}H_{12}N_4O_3S$	1220-83-3	280.3	0.70	1.98, 5.96 [g]
	Sulfaphenazole	SPZ	$C_{15}H_{14}N_4O_2S$	526-08-9	314.4	1.52	2.43, 6.90 [e]
	Sulfapyridine	SPD	$C_{11}H_{11}N_3O_2S$	144-83-2	249.3	0.35	2.90, 8.54 [b]
	Sulfaquinoxaline	SQX	$C_{14}H_{12}N_4O_2S$	59-40-5	300.4	1.68	1.86, 5.56 [g]
	Sulfathiazole	STZ	$C_9H_9N_3O_2S_2$	72-14-0	255.3	0.05	2.20, 7.24 [b]
	Sulfisoxazole	SSZ	$C_{11}H_{13}N_3O3_S$	127-69-5	267.3	1.01	1.66, 4.71 g
	Sulfacetamide	SCT	$C_8H_{10}N_2O_3S$	144-80-9	214.2	-0.96	5.4 [l]
	Sulfaguanidine	SGD	$C_7H_{10}N_4O_2S$	57-67-0	214.2	-0.33	1.55,
	Sulfisomidine	SSM	$C_{12}H_{14}N_4O_2S$	515-64-0	278.3	-0.33	NA
	Sulfamoxole	SMO	$C_{11}H_{13}N_3O_3S$	729-99-7	267.3	1.03	NA

Class	Antibiotic	Abbreviation	Molecular formula	CAS No.	Molecular weight (g/mol)	Log Kow [a]	pKa
	Trimethoprim	TMP	$C_{14}H_{18}N_4O_3$	738-70-5	290.3	0.91	3.23, 6.76 [f]
QNs	Ciprofloxacin	CIP	$C_{17}H_{18}FN_3O_3$	85721-33-1	331.3	0.28	3.10, 6.14, 8.70, 10.58 [f]
	Danofloxacin	DAN	$C_{19}H_{20}FN_3O_3$	112398-08-0	357.4	0.44	6.07, 8.56 [i]
	Difloxacin	DIF	$C_{21}H_{19}F_2N_3O_3$	98106-17-3	399.4	0.89	5.66, 7.24 [i]
	Enoxacin	ENO	$C_{15}H_{17}FN_4O_3$	74011-58-8	320.3	-0.20	5.50, 8.59 [e]
	Enrofloxacin	ENR	$C_{19}H_{22}FN_3O_3$	93106-60-6	359.4	0.70	3.86, 6.19, 7.59, 9.86 [f]
	Fleroxacin	FLE	$C_{17}H_{18}F_3N_3O_3$	79660-72-3	369.3	0.24	5.44, 6.06 [e]
	Flumequine	FLU	$C_{14}H_{12}FNO_3$	42835-25-6	261.3	1.60	6.50 [c]
	Lomefloxacin	LOM	$C_{17}H_{19}F_2N_3O_3$	98079-51-7	351.3	-0.30	5.00, 5.87, 9.23 [b]
	Nalidixic Acid	NDA	$C_{12}H_{12}N_2O_3$	389-08-2	232.2	1.59	6.01 [i], 8.60 [c]
	Norfloxacin	NOR	$C_{16}H_{18}FN_3O_3$	70458-96-7	319.3	-1.03	3.11, 6.10, 8.60, 10.56 [f]
	Ofloxacin	OFL	$C_{18}H_{20}FN_3O_4$	82419-36-1	361.4	-0.39	5.97, 8.28 [d]
	Orbifloxacin	ORB	$C_{19}H_{20}F_3N_3O_3$	113617-63-3	395.4	2.37	NA
	Oxolinic Acid	OLA	$C_{13}H_{11}NO_5$	14698-29-4	261.2	0.94	6.90 [d]
	Pefloxacin	PEF	$C_{17}H_{20}FN_3O_3$	70458-92-3	333.4	0.27	5.66, 6.47 [e]
	Sarafloxacin	SAR	$C_{20}H_{17}F_2N_3O_3$	98105-99-8	385.4	1.07	5.60, 8.20 [c]
	Sparfloxacin	SPA	$C_{19}H_{22}F_2N_4O_3$	110871-86-8	392.4	2.50	5.75, 8.79 [e]
TCs	4-Epianhydroustetracycline hydrochloride	EATC	$C_{22}H_{22}N_2O_7 \cdot HCl$	4465-65-0	462.9	NA	NA
	4-Epichlortetracycline	ECTC	$C_{22}H_{23}ClN_2O_8$	14297-93-9	478.9	0.33 [m]	NA
	4-Epioxytetracycline	EOTC	$C_{22}H_{24}N_2O_9$	14206-58-7	460.4	-1.50 [m]	NA
	4-Epitetracycline hydrochloride	ETC	$C_{22}H_{25}ClN_2O_8$	23313-80-6	480.9	-1.33	NA
	Anhydrochlortetracycline	ACTC	$C_{22}H_{21}ClN_2O_7$	4497-08-9	460.9	2.24 [m]	NA
	Anhydrotetracycline	ATC	$C_{22}H_{22}N_2O_7$	1665-56-1	426.4	0.63	NA

Class	Antibiotic	Abbreviation	Molecular formula	CAS No.	Molecular weight (g/mol)	Log Kow [a]	pKa
	Chlorotetracycline	CTC	$C_{22}H_{23}ClN_2O_8$	57-62-5	478.9	-0.62	3.30, 7.55, 9.15 [b]
	Demeclocycline	DCTC	$C_{21}H_{21}ClN_2O_8$	127-33-3	464.9	-1.14	8.23 [e]
	Doxycycline	DC	$C_{22}H_{24}N_2O_8$	564-25-0	444.4	-0.02	7.75 [e]
	4-Epianhydrochlortetracycline	EACTC	$C_{22}H_{21}ClN_2O_7$	81163-11-3	460.9	1.28	NA
	Isochlortetracycline	ICTC	$C_{22}H_{23}ClN_2O_8$	514-53-4	478.9	1.99	NA
	Oxytetracycline	OTC	$C_{22}H_{24}N_2O_9$	79-57-2	460.4	-0.90	3.27, 7.32, 9.11 [d]
	Tetracycline	TC	$C_{22}H_{24}N_2O_8$	60-54-8	444.4	-1.30	3.30, 7.68, 9.69 [d]
MLs	Anhydroerythromycin	AETM	$C_{37}H_{65}NO_{12}$	23893-13-2	715.9	4.34	NA
	Azithromycin	AZM	$C_{38}H_{72}N_2O_{12}$	83905-01-5	749.0	4.02	8.74, 9.45 [c]
	Clarithromycin	CTM	$C_{38}H_{69}NO_{13}$	81103-11-9	747.9	3.16	8.99 [c]
	Erythromycin	ETM	$C_{37}H_{67}NO_{13}$	114-07-8	733.9	3.06	8.90 [f]
	Roxithromycin	RTM	$C_{41}H_{76}N_2O_{15}$	80214-83-1	837.0	2.75	9.17 [f]
	Tilmicosin	TIL	$C_{46}H_{80}N_2O_{13}$	108050-54-0	869.2	3.80	8.18, 9.56 [j]
	Tylosin	TYL	$C_{46}H_{77}NO_{17}$	1401-69-0	916.1	1.63	7.73 [c]
	Josamycin	JSM	$C_{42}H_{69}NO_{15}$	16846-24-5	828.0	3.16	7.9, 12.67 [e]
	Leucomycin	LM	$C_{39}H_{65}NO_{14}$	18361-45-0	771.9	2.58	NA
	Spiramycin	SPM	$C_{43}H_{74}N_2O_{14}$	8025-81-8	843.1	1.87	7.88, 9.28 [c]
β-Ls	Amoxicillin	AMX	$C_{16}H_{19}N_3O_5S$	26787-78-0	365.4	0.87	2.23, 7.43
	Ampicillin	AMP	$C_{16}H_{19}N_3O4S$	69-53-4	349.4	1.35	2.50, 7.30 [c]
	Cefadroxil	CED	$C_{16}H_{17}N_3O_5S$	50370-12-2	363.4	-0.40	3.45, 7.43 [e]
	Cefazolin	CEZ	$C_{14}H_{14}N_8O_4S_3$	25953-19-9	454.5	-0.58	3.6 [c]
	Cefotaxime	CFT	$C_{16}H_{17}N_5O_7S_2$	63527-52-6	455.5	0.64	3.18, 4.15 [e]
	Cephalexin	CEL	$C_{16}H_{17}N_3O_4S$	15686-71-2	347.4	0.65	5.20, 7.30 [c]
	Cephradine	CER	$C_{16}H_{19}N_3O_4S$	38821-53-3	349.4	0.41	2.60,7.30 [c]

Class	Antibiotic	Abbreviation	Molecular formula	CAS No.	Molecular weight (g/mol)	Log Kow [a]	pKa
	Cloxacillin	CLX	$C_{19}H_{17}ClN_3O_5S$	61-72-3	435.9	2.48	2.78
	Desacetylcefotaxime	DSX	$C_{14}H_{15}N_5O_6S_2$	66340-28-1	413.4	0.88	NA
	Dicloxacillin	DLX	$C_{19}H_{16}Cl_2N_3O_5S$	3116-76-5	470.4	2.91	2.80 [c]
	Nafcillin sodium salt	NAF	$C_{21}H_{21}N_2O_5S \cdot Na$	985-16-0	436.5	-1.07	3.31 [e]
	Oxacillin	OXA	$C_{19}H_{19}N_3O_5S$	66-79-5	401.4	2.38	2.72
	Penicillin G	PCG	$C_{16}H_{18}N_2O_4S$	61-33-6	334.4	1.83	2.74 [c]
	Penicillin V	PCV	$C_{16}H_{18}N_2O_5S$	87-08-1	350.4	2.09	2.79 [e]
	Cefapirin	CEP	$C_{17}H_{17}N_3O_6S_2$	21593-23-7	423.5	-1.15	2.74, 5.13[n]
PEs	Lasalocid	LA	$C_{34}H_{54}O_8$	25999-20-6	590.8	6.74	NA
	Maduramycin	MAD	$C_{47}H_{83}NO_{17}$	84878-61-5	934.2	NA	NA
	Monensin	MON	$C_{36}H_{62}O_{11}$	17090-79-8	670.9	5.43	4.2
	Nigericin	NIG	$C_{40}H_{68}O_{11}$	28380-24-7	725.0	NA	NA
	Salinomycin	SAL	$C_{42}H_{70}O_{11}$	53003-10-4	751.0	8.53	6.4
LMs	Clindamycin	CDM	$C_{18}H_{33}ClN_2O_5S$	18323-44-9	425.0	2.16	7.55, 12.16 [e]
	Lincomycin	LCM	$C_{18}H_{34}N_2O_6S$	154-21-2	406.5	0.56	7.60 [c]
Internal standards	Sulfamethoxazole-d4	SMX-d4	$C_{10}H_7D_4N_3O_3S$	1020719-86-1	257.3	NA	NA
	Tetracycline-d6	TC-d6	$C_{22}H_{18}D_6N_2O_8$	NA	450.5	NA	NA
	Ciprofloxacin-d8	CIP-d8	$C_{17}H_{10}D_8FN_3O_3$	1130050-35-9	339.39	NA	NA
	Roxithromycin-d7	RTM-d7	$C_{41}H_{69}D_7N_2O_{15}$	NA	450.5	NA	NA
	Amoxicillin-d4	AMX-d4	$C_{16}H_{15}D_4N_3O_5S$	26787-78-0	365.4	NA	NA

[a] logK_{ow} (octanol-water coefficient) from ChemIDPlus Advanced (http://chem.sis.nlm.nih.gov/chemidplus/) and PubChem (https://pubchem.ncbi.nlm.nih.gov/), U.S. National Library of Medicine

[b] (Chen and Zhou 2014)[4]

[c] TOXNET, Toxicology Data Network (https://toxnet.nlm.nih.gov/), U.S. National Library of Medicine

[d] (Tolls, 2001)

[e] Drugbank database Version 5.0 (https://www.drugbank.ca/), University of Alberta and Metabolomics Innovation Centre, Canada

[f] (Qiang and Adams, 2004)[5]; [g] (Lin et al., 1997)[6]; [h] (Geiser et al., 2005)[7]; [i] (Jimenez-Lozano et al., 2002)[8]; [j] (McFarland et al., 1997)[9]; [k] (Zhao et al., 2015)[10]; [l] (Zhou et al., 2016)[11]

[m] Predicted data is generated using the ACD/Labs Percepta Platform - PhysChem Module (http://www.chemspider.com/)

[n] (Ribeiro, Alyson, R, et al., 2017)[12]

Table S2 Detailed parameters for UHPLC.

Antibiotic	Time (min)	Elute A: methanol (%)	Elute B: 0.1% (v/v) formic acid (%)	Flow rate (mL/min)	Injection volume (μL)
SAs,	0	20	80	0.20	5
SMX-d4,	2.0	20	80		
	2.5	25	75		
	4.8	40	60		
	7.0	95	5		
	7.1	20	80		
	10.0	20	80		
MLs,	0	30	70	0.25	5
LMs	1.0	70	30		
RTM-d7	2.0	70	30		
	5.0	80	20		
	5.1	30	70		
	7.0	30	70		
TCs	0	20	80	0.25	5
TC-d6,	0.5	20	80		
	6.0	96	5		
	7.0	95	5		
	7.1	20	80		
	9.0	20	80		
FQs	0	20	80	0.25	5
CIP-d8,	1.0	20	80		
	3.0	40	60		
	6.0	95	5		
	7.0	95	5		
	7.1	20	80		
	9.0	20	80		
β-Ls,	0	20	80	0.25	5
AMX-d4	1.0	20	80		
	2.5	25	75		
	4.0	50	50		
	6.0	70	30		
	10.5	95	5		
	11.0	95	5		
	11.1	20	80		
	13.0	20	80		
PEs	0	90	10	0.25	5
RTM-d7	6	90	10		

Table S3 Detailed parameters for MS.

Antibiotic	Sheath gas (L/min)	Auxiliary gas (L/min)	Spray voltage (V)	ESI
SAs, SMX-d4	4.09	5.08	3500	+
MLs, LMs, RTM-d7	3.62	2.42	3500	+
TCs, TC-d6	4.78	4.3	3000	+
FQs, CIP-d8	3.62	3.41	3500	+
β-Ls, AMX-d4	4.58	6.4	3000	+
PEs, RTM-d7	4.09	5.08	3500	+

Table S4 Water quality in different sampling sites from Huangshui River.

Sampling sites	WT (^oC)	pH	DO (mg/L)	TOC (mg/L)	NH_4-N (mg/L)	NO_3-N (mg/L)	NO_2-N (mg/L)	TN (mg/L)	TP (mg/L)
Wet									
T1	22.4	8.58	4.80	1.79	0.189	1.645	0.071	1.387	0.012
T2	19.2	8.39	5.71	1.45	0.217	1.496	0.049	1.308	< 0.01
T5	17.8	8.33	5.19	1.80	0.106	2.044	0.252	1.901	0.010
T6	19.0	8.19	1.18	2.95	1.001	3.047	0.718	3.290	< 0.01
T7	21.0	8.40	4.26	1.58	0.441	3.562	1.014	3.464	< 0.01
M1	19.0	8.51	5.32	32.00	0.241	1.647	0.129	1.373	< 0.01
M2	17.9	8.49	5.67	2.01	0.268	1.915	0.111	1.695	0.015
M3	18.9	8.40	5.00	2.19	2.265	2.334	0.250	2.802	< 0.01
M5	19.0	8.40	5.04	2.05	2.203	2.728	0.550	3.156	< 0.01
M6	16.3	8.30	3.25	2.26	0.945	2.487	0.555	2.697	< 0.01
M7	22.2	8.30	5.20	2.35	1.219	2.652	0.729	2.871	< 0.01
M10	17.4	8.19	5.85	2.17	1.678	2.816	0.686	3.294	0.010
W1	19.9	7.66	3.95	6.51	12.726	0.591	0.297	14.128	0.018
W3	-	-	-	4.04	0.303	10.842	0.025	10.115	0.015
Dry									
T1	8.3	8.16	8.09	3.07	0.396	1.755	0.281	2.124	< 0.01
T2	7.7	8.28	8.39	2.44	0.524	1.016	0.173	1.420	< 0.01
T3	13.7	8.47	8.00	2.58	0.354	2.581	0.773	4.752	0.06
T4	12.6	8.41	7.77	3.01	1.062	2.462	0.773	4.920	0.05
T5	11.1	8.26	7.96	4.28	0.632	1.825	0.810	1.995	< 0.01
T6	12.2	8.15	8.11	6.24	0.248	5.473	1.116	4.699	< 0.01
T7	9.7	8.26	8.04	2.50	0.214	3.754	0.514	3.103	< 0.01
M1	8.2	8.37	7.76	0.33	0.612	1.755	0.352	2.296	< 0.01
M2	8.9	8.35	8.06	3.31	1.111	1.763	0.631	2.407	< 0.01
M3	10.9	8.23	7.69	2.70	0.733	1.692	0.361	2.759	< 0.01
M4	12.1	8.37	7.42	2.75	2.497	3.774	1.009	6.509	0.05
M5	9.2	8.31	8.00	3.55	1.495	2.976	1.645	3.824	< 0.01
M6	15.9	8.45	9.34	4.12	0.949	3.377	2.885	4.382	< 0.01
M7	9.7	8.28	7.39	1.90	1.475	2.346	1.475	3.257	< 0.01
M8	10.8	8.26	7.24	1.17	1.084	3.816	1.699	2.896	< 0.01
M9	12.2	8.09	7.33	3.22	1.165	4.656	2.193	4.485	0.01
M10	9.7	8.18	8.03	5.65	1.455	3.182	1.852	3.575	< 0.01
W1	15.2	6.95	6.49	3.87	0.570	16.220	0.370	17.400	< 0.01
W2	15.6	7.70	5.00	2.33	13.600	10.315	1.875	0.580	0.06
W3	14.0	7.24	7.01	7.36	0.640	8.313	0.577	8.900	0.01
W4	11.2	7.48	7.21	2.94	1.117	11.353	0.084	24.424	0.07
W5	11.6	7.58	7.35	2.81	0.209	8.092	0.109	18.376	0.05
W6	14.9	7.04	7.21	4.44	0.807	14.556	2.050	13.198	0.01

LOQ: NO_2-N: 0.003mg/L, NH_4-N: 0.025 mg/L, NO_3-N: 0.08 mg/L, TN: 0.05 mg/L, TP: 0.01 mg/L.

Table S5 Sediment quality in different sampling sites from Huangshui River.

Sampling sites	pH	NH_4-N (mg/kg)	NO_3-N (mg/kg)	NO_2-N (mg/kg)	TN (mg/kg)	TP (mg/kg)	SOC (%)
Wet							
T1	8.72	6.19	2.13	2.94	2530	67	0.61
T2	8.41	4.90	0.47	ND	3480	110	0.31
T5	8.57	7.02	1.71	ND	2060	75	0.88
T6	8.24	19.50	1.73	ND	8700	172	1.81
T7	9.24	1.08	<1	ND	1580	42	1.27
M1	8.83	2.42	1.71	ND	1270	74	0.35
M2	9.08	3.70	10.80	ND	949	137	0.35
M3	9.02	5.23	24.80	ND	1110	93	1.11
M5	9.16	1.59	13.70	ND	2530	58	0.63
M6	8.41	13.40	2.13	ND	3640	100	1.66
M7	9.20	5.00	1.10	ND	791	230	0.37
M10	8.70	1.34	1.43	ND	1420	139	0.57
Dry							
T1	7.65	0.58	10.3	<0.6	1740	328	0.07
T2	7.85	<0.4	8.26	<0.6	950	324	0.10
T3	7.38	0.69	29	ND	1290	348	0.11
T4	7.67	0.52	17.5	<0.6	470	311	0.04
T5	7.51	<0.4	49.8	ND	610	291	0.06
T6	7.44	0.4	10.2	ND	1510	295	0.15
T7	7.88	0.48	49.1	ND	470	336	0.06
M1	7.74	0.62	<1	<0.6	710	272	0.13
M2	8.2	<0.4	8.24	ND	430	252	0.05
M3	7.81	<0.4	11.7	ND	530	286	0.10
M4	8.36	0.48	16	ND	1580	200	0.07
M5	7.77	0.56	9.9	ND	410	222	0.13
M6	7.62	0.81	2.07	<0.6	1770	328	0.24
M7	7.82	<0.4	11.1	ND	430	220	0.13
M8	7.84	<0.4	<1	ND	460	308	0.07
M9	7.63	0.63	14.5	<0.6	1770	432	0.10
M10	8.03	0.52	3.44	ND	180	253	0.06

ND, not detected

LQ@: NH_4-N: 0.4 mg/kg, NO_3-N: 1 mg/kg, NO_2-N: 0.6mg/kg, TN: 48 mg/kg, TP: 10 mg/kg.

Table S6 Predicted no-effect-concentrations for resistance selection ($PNEC_{AMR}$) from previous studies (ng/L).

Class	Antibiotic	$PNEC_{AMR}$	Class	Antibiotic	$PNEC_{AMR}$
SAs	SDZ	5000[a]		OFL	40[a]
	SMX	16000[b]		NOR	150[a]
	TMP	1000 [a]		ENR	64[b]
MLs	AZM	150[a]		PEF	8000[b]
	CTM	40[a]	TCs	OTC	500[b]
	RTM	150[a]		TC	300[a]
	ETM	40[a]		DC	300[a]
	TYL	4000[b]	β-Ls	CLX	125[b]
	TIL	1000[b]		AMP	75 [a]
	SPM	500[b]		CFT	125[b]
QNs	FLU	250[b]	LMs	CDM	500[a]
	NDA	16000[b]		LCM	2000[b]

[a] (Kümmerer and Hnninger, 2003)[13]; [b] (Bengtsson-Palme and Larson, 2016)[14]

Table S7 Summary of chronic toxicity data calculated by ECOSAR (v2.0, USEPA).

Class	Antibiotic	Green algae (mg/L)	Daphnid (mg/L)	Fish (mg/L)	Class	Antibiotic	Green algae (mg/L)	Daphnid (mg/L)	Fish (mg/L)
SAs	SGD	17.74	0.21	2.78		PEF	52.95	9.22	186.29
	SDZ	14.89	0.15	1.50		OLA	51.49	0.59	2.85
	SMX	10.32	0.09	0.64		SPA	44.90	8.27	147.44
	TMP	0.77	0.08	1.79	TCs	OTC	1.69	1.85	40.39
	SMZ	8.61	0.08	0.53		EACTC	0.24	0.09	0.44
	SPD	9.73	0.08	0.60		TC	0.79	0.59	7.29
	SMM	13.10	0.12	0.96		ECTC	1.01	0.82	11.66
	SFM	16.08	0.16	1.54		ICTC	0.18	0.06	0.21
	SCP	12.67	0.11	0.87		ETC	1.75	1.90	41.25
	STZ	8.24	0.07	0.51		DC	0.80	0.60	7.58
	SPZ	4.39	0.05	0.26		ATC	0.30	0.14	0.83
	SDM	6.30	0.07	0.38		EATC	0.30	0.14	0.83
MLs	AETM	0.12	0.07	0.10		EOTC	1.69	1.85	40.39
	AZM	0.69	0.29	0.82		ACTC	0.24	0.09	0.44
	CTM	0.76	0.31	0.93		CTC	0.63	4.17	20.20
	RTM	1.65	0.60	2.30	β-Ls[a]	CLX	1.13	1.38	0.06
	ETM	1.65	0.60	2.30		PCG	3.44	7.68	0.20
	JSM	0.87	0.04	0.10		PCV	3.53	7.81	0.21
	TYL	24.90	5.84	36.24		AMP	5.09	1.32	10.80
	TIL	1.74	1.02	0.32		CFT	4.31	0.14	0.39
	LM	1.96	0.06	0.18		NAF	0.60	0.57	0.03
	SPM	6.43	1.87	0.51		CER	25.6	5.08	76.50
QNs[a]	FLU	12.28	7.44	17.45	PEs	MON	5.20	0.84	0.65
	NDA	49.99	46.57	153.84		MAD	5.32	1.16	9.30
	OFL	67.49	11.44	245.68		LA	0.03	0.05	0.64
	NOR	70.25	11.59	264.72	LMs	CDM	2.64	0.79	0.22
	ENR	16.71	3.58	36.73		LCM	20.18	6.88	1.26

[a] Predicted values were divided by 10 (Li et al., 2018)[2].

Table S8 Summary of information on the STPs.

STPs	Average treatment capacity (m^3/d)	Technology	Operation date	Effluent standard
W1	82300	A/A/O+JS-BC biological treatment process	2002 (upgrading and construction In 2016)	I A
W2	42500	OD (Oxidation ditch)	2006	II
W3	98000	A/A/O-MBBR	2010 (upgrading and construction in 2017)	I A
W4	27600	Multistage AO & Chemical phosphorus removal	2015	I A
W5	15000	Multistage AO & UV disinfection	2015	I A
W6	100000	Multistage AO & UV disinfection	2019	I A

A/A/O-MBBR: anaerobic/anoxic/oxic; MBBR: Moving bed biofilm reactor; AO: anaerobic /oxic; UV: ultraviolet

Table S9 Summary of antibiotic concentrations in urban river water in different regions of China and other countries in the world.

Region	Antibiotics	Range (ng/L)	Median (ng/L)	Mean (ng/L)	References
Bohai Rim	SDZ	ND−2396	14.4	98.3	15, 16, 17, 18, 19, 20, 21, 22, 23, 25, 26, 27, 28, 29, 30, 31, 32, 33, 34, 35, 36, 38, 39, 40, 41
	SMZ	ND−3900	4.30	121	
	SMX	ND−145290	56.6	2049	
	OTC	ND−361107	10.6	3735	
	TC	ND−387000	3.30	3216	
	OFL	ND−11735	13.0	251	
	NOR	ND−1380	28.4	95.3	
	CIP	ND−1399	19.2	68.5	
	ENR	ND−53969	0.70	516	
	ETM	ND−4200	14.9	137	
	RTM	ND−3700	37.8	295	
	CTM	ND−96.9	2.20	9.80	
Yangtze River Delta	SDZ	ND−320	11.0	33.3	4, 10, 42, 43, 44, 45, 46, 47, 48, 49, 50, 52, 53, 54, 55, 56, 57
	SMZ	ND−930	5.03	68.0	
	SMX	0.10−765	8.71	46.4	
	OTC	ND−2260	6.38	49.4	
	TC	ND−1000	32.4	81.5	
	OFL	ND−254	4.17	10.4	
	NOR	ND−71.0	1.54	6.76	
	CIP	ND−57.0	1.40	6.31	
	ENR	ND−33.0	1.70	4.83	
	ETM	0.02−543	14.8	69.2	
	RTM	ND−33.1	1.20	2.75	
	CTM	0.04−67.3	2.26	8.82	
Southeast Coastal Economic Zone	SDZ	ND−344	3.41	36.8	34, 58, 59, 60, 61, 62, 63, 64,65, 66, 6667, 68, 68, 69, 70, 71, 72, 73, 74, 75, 76, 77
	SMZ	ND−2479	16.8	94.2	
	SMX	ND−1697	9.41	79.4	
	OTC	ND−457	7.80	47.6	
	TC	ND−384	14.9	29.8	
	OFL	ND−779	6.38	52.2	
	NOR	ND−6620	6.70	391	
	CIP	ND−415	2.50	85.8	
	ENR	ND−2.54	0.75	0.82	
	ETM	ND−891	2.50	117	
	RTM	ND−2260	3.12	71.2	
	CTM	ND−603	0.57	32.3	
Northeast China	SDZ	ND−13.9	ND	0.65	78, 79, 80, 81
	SMZ	ND−0.39	ND	0.02	
	SMX	ND−73.1	0.11	8.04	
	OTC	ND−0.02	ND	ND	
	TC	ND−0.27	ND	0.02	
	OFL	ND−50.8	0.38	5.13	
	NOR	ND−2.40	0.45	0.95	
	CIP	ND−1.40	0.08	0.26	
	ENR	ND−35.9	1.35	6.85	
	ETM	0.15−34.7	3.70	8.88	
	RTM	0.20−11.5	3.89	5.20	
	CTM	ND−4.17	1.25	1.81	
Central and West China	SDZ	0.20−379	3.33	18.6	35, 82, 83, 84, 85, 86, 87, 88, 89, 90, 91, 92, 93
	SMZ	ND−464	2.34	14.0	
	SMX	ND−1336	12.1	48.0	
	OTC	ND−1500	6.43	112	

Region	Antibiotics	Range (ng/L)	Median (ng/L)	Mean (ng/L)	References
	TC	ND−6800	5.64	230	
	OFL	ND−264	9.30	51.5	
	NOR	ND−327	11.6	43.7	
	CIP	0.08−96.0	6.29	10.7	
	ENR	ND−351	5.21	30.4	
	ETM	ND−2910	57.0	289	
	RTM	ND−236	6.98	29.1	
	CTM	ND−103	1.10	7.83	
Huangshui River	SDZ	0.22−58.9	4.17	11.0	This study
	SMZ	ND−9.99	1.33	2.28	
	SMX	1.17−552	46.6	90.4	
	OTC	ND−24.3	2.45	5.14	
	TC	ND−1.93	0.56	0.66	
	OFL	ND−8.33	0.24	0.94	
	NOR	–[a]	–	–	
	CIP	–	–	–	
	ENR	–	–	–	
	ETM	0.30−16.6	2.82	4.16	
	RTM	0.12−48.7	3.71	7.10	
	CTM	0.06−7.75	1.03	1.43	
Urban rivers in Japan	SMZ	ND−9.80	ND	1.97	94
	SMX	11.9−33.9	21.6	21.7	
	RTM	5.5−27.8	16.1	15.5	
	CTM	50.7−233	110	121	
Urban rivers in Vietnam	SMZ	<LOQ[b]−128	53.0	–	95
	SMX	310−15591	7631	–	
	OTC	<LOQ−116	<LOQ	–	
	TC	<LOQ−635	101	–	
	OFL	45−2867	272	–	
	CIP	<LOQ−3035	302	–	
	ENR	55−2869	226	–	
	ETM	<LOQ−48517	5542	–	
	CTM	7−3944	700	–	
Urban rivers in Brazil	SMX	ND−1800	755	–	96
	CIP	ND−70	ND	–	
	NOR	ND−130	10	–	
Urban rivers in USA	SMX	ND−576	0.5	–	97
Urban rivers in Australia	SMX	ND−2000	8	–	98
	OTC	ND−100	ND	–	
	TC	ND−80	ND	–	
	NOR	ND−1150	30	–	
	CIP	ND−1300	ND	–	
	ENR	ND−300	ND	–	
	ETM	<LOQ	<LOQ	–	
	RTM	ND−350	9	–	

[a] – data not available, [b] LOQ, limit of quantification.

Table S10 Summary of population and gross domestic product (GDP) in different regions of China.[a, b]

Region	Province/City	Population (person/km^2)	COD discharge (t/km^2·a)	GDP (RMB per capita)
Bohai Rim	Beijing	1312	4.98	140211
	Tianjin	1311	7.78	120711
	Hebei	404	2.60	47772
	Shandong	639	3.31	76267
	Liaoning	294	1.71	58008
Yangtze River Delta	Shanghai	155	0.905	134982
	Jiangsu	786	7.27	115168
	Zhejiang	554	4.04	98643
	Anhui	453	3.55	47712
Southeast coastal economic zone	Guangdong	651	5.74	86412
	Fujian	321	3.21	91197
Northeast China	Heilongjiang	85.8	0.564	43274
	Jilin	142	0.917	55611
Central and west China	Shanxi	237	1.25	45328
	Henan	576	2.58	50152
	Hubei	319	2.80	66616
	Chongqing	377	3.07	65933
	Guizhou	204	1.55	41244
	Yunnan	123	0.839	37136
	Guangxi	207	1.92	41489
	Shaanxi	188	0.955	63477
	Xinjiang	15.2	0.121	49475
	Qinghai	8.66	0.0825	47689

[a] Population, COD, and GDP data source: http://data.cnki.net/yearbook/Single /N2019110002

[b] Area data source: http://xzqh.mca.gov.cn/map

Table S11 Toxicity data of antibiotics to bacteria.

Class	Antibiotic	Bacteria species	Duration	EC_{50} (mg/L)	Reference
SAs	SGD	*V. fischeri*	30 min	>50	99
	SDZ	*V. fischeri*	30 min	>25	99
	SMX	*V. fischeri*	5 min	52.92	100
			5 min	74.2	101
			15 min	43.56	100
			15 min	78.1	101
			30 min	23.3	102
			30 min	>100	99
			30 min	140	103
			30 min	>84	104
			24 h	1.77	103
			**NOEC**	**93.01**	**105**
	TMP	*V. fischeri*	**5 min**	**165.1**	**101**
			15 min	176.7	101
	SMZ	*V. fischeri*	**5 min**	**303.0**	**101**
			15 min	344.7	101
			30 min	>50	99
	SPD	*V. fischeri*	30 min	>50	99
	STZ	*V. fischeri*	5 min	>1000	101
			15 min	>1000	101
			30 min	>50	99
MLs	CTM	*V. fischeri*	5 min	12.65	100
			**15 min**	**12.08**	**100**
			30 min	>100	102
	RTM	*V. fischeri*	5 min	>1000	106
			15 min	>1000	106
	ETM	*V. fischeri*	30 min	>100	102
			**NOEC**	**82.03**	**105**
QNs	FLU	*V. fischeri*	30 min	11	107
			30 min	12.10	108
			24 h	0.019	109
			**NOEC**	**0.31**	**107**
	OFL	*V. fischeri*	30 min	>90	104
			30 min	>100	102
			24 h	0.014	109
			**NOEC**	**0.00113**	**109**
	NDA	*V. fischeri*	24 h	0.200	109
			**NOEC**	**0.074**	**105**
	OLA	*V. fischeri*	24 h	0.022	109
			**NOEC**	**0.00073**	**105**
TCs[a]	OTC	*V. fischeri*	5 min	235.4	110
			15 min	87.0	110

Class	Antibiotic	Bacteria species	Duration	EC_{50} (mg/L)	Reference
			15 min	70	111
			30 min	21.0	107
			30 min	64.5	102
			30 min	108	112
			30 min	121.0	108
			24 h	0.081	105
			**NOEC**	**0.02**	**105**
			NOEC	2.5	107
		E. coli	24 h	0.143	113
		B. subtilis	24 h	0.060	113
	CTC	*V. fischeri*	5 min	>20.0	110
			15 min	13.0	110
			24 h	10.03	105
			**NOEC**	**0.04**	**105**
		E. coli	24 h	0.158	113
		B. subtilis	24 h	0.014	113
	TC	*V. fischeri*	**24 h**	**0.025**	**114**
		E. coli	24 h	0.133	113
		B. subtilis	24 h	0.036	113
	DC	*E. coli*	24 h	0.058	113
		B. subtilis	**24 h**	**0.009**	**113**
		V. fischeri	15 min	32	111
β-Ls	AMP	*V. fischeri*	5 min	1056	110
			15 min	2627	110
			24 h	163	114
			**NOEC**	**65.03**	**105**
LMs	CDM	*V. fischeri*	24 h	92.03	105
			**NOEC**	**1.80**	**105**
	LCM	*V. fischeri*	**15 min**	**4300**	**115**
		V. fischeri	30 min	>100	102

[a] Values of hydrous and anhydrous forms of antibiotics were assumed to be identical. Values of isomers were assumed to be identical.

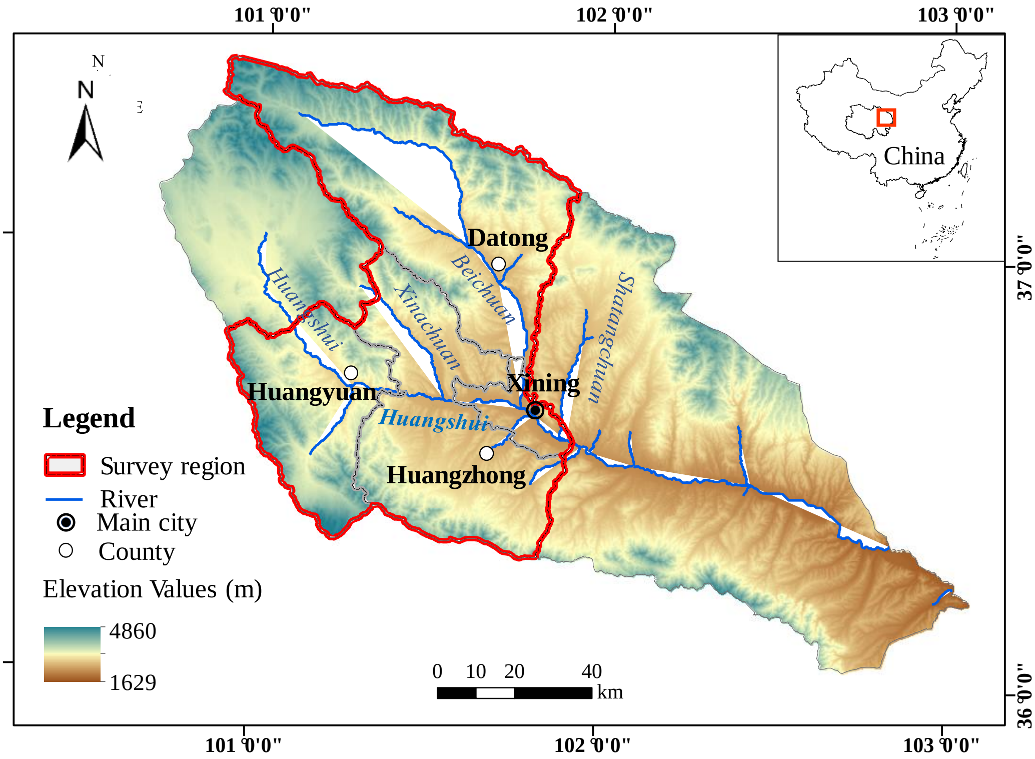

Figure S1. Location of the Huangshui River Basin and the area of Xining City. Map created using ArcGIS 10.2–http://developers.arcgis.com; Image from http://www.gscloud.cn.

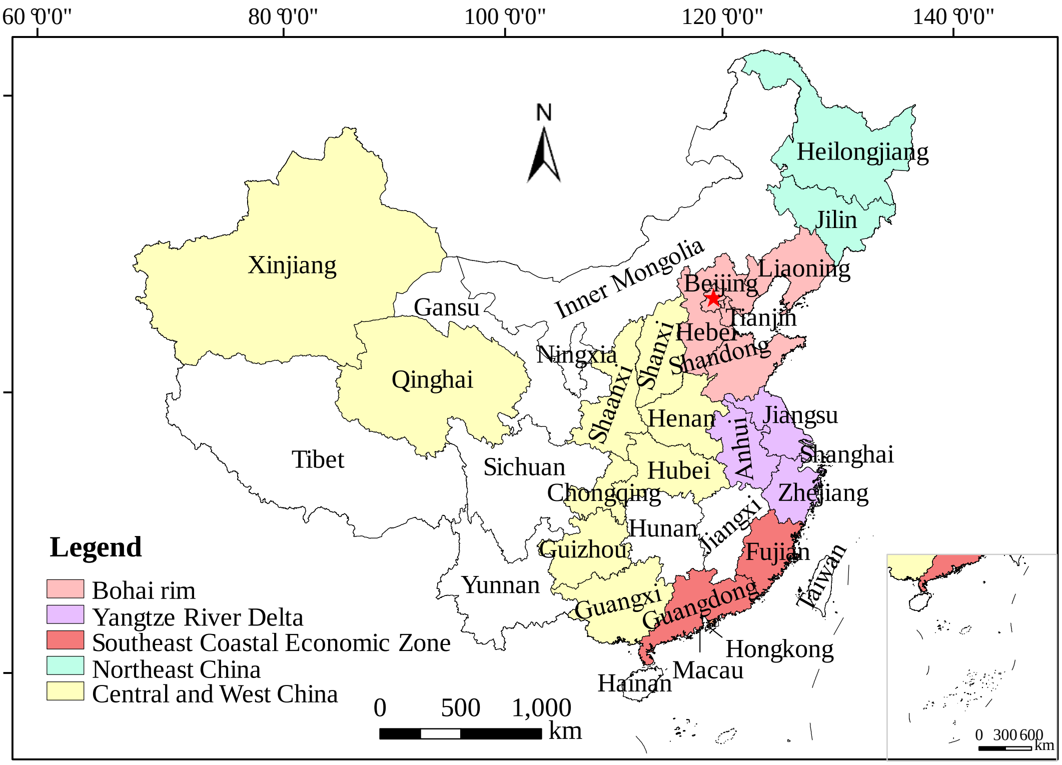

(a)

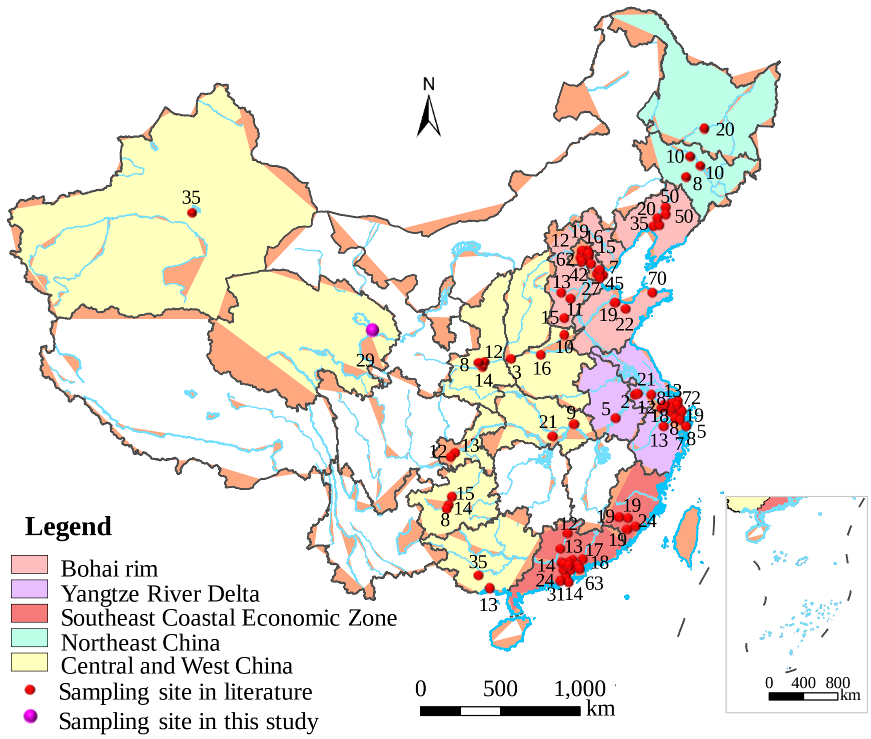

(b)

Figure S2. Different regions of China divided according to the economic development and geographical location of different provinces (The white color in the map means no data in this province) (a) and sampling sites in urban rivers of this and previous studies (The number of the sapling sites is labelled) (b). Maps created using ArcGIS 10.2 – http://developers.arcgis.com; Image from http://www.resdc.cn/Default.aspx.

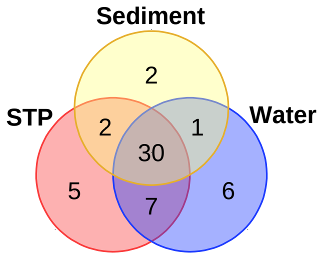

Figure S3. Venn plots of detected antibiotics in water, sediments, and E-STPs.

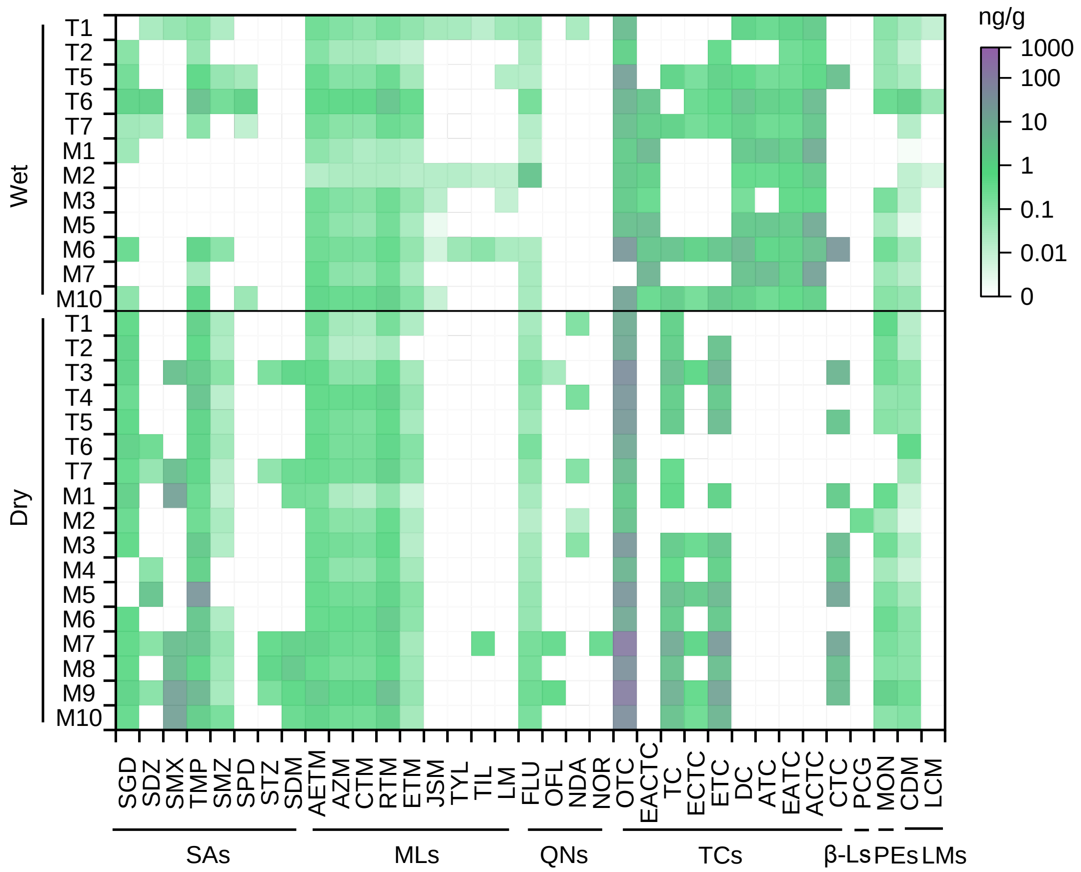

Figure S4. Antibiotic concentrations in sediments from the Huangshui River.

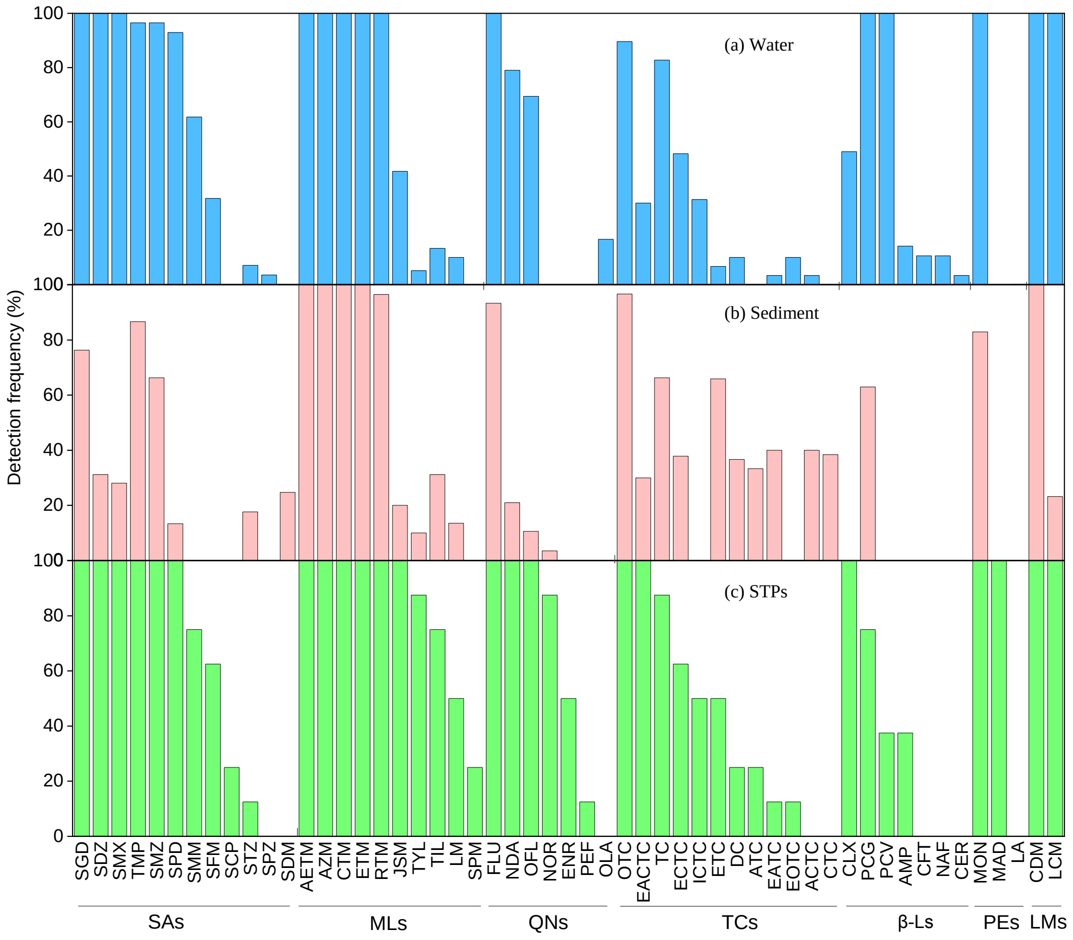

Figure S5. Detection frequencies of antibiotics in water, sediments, and E-STPs.

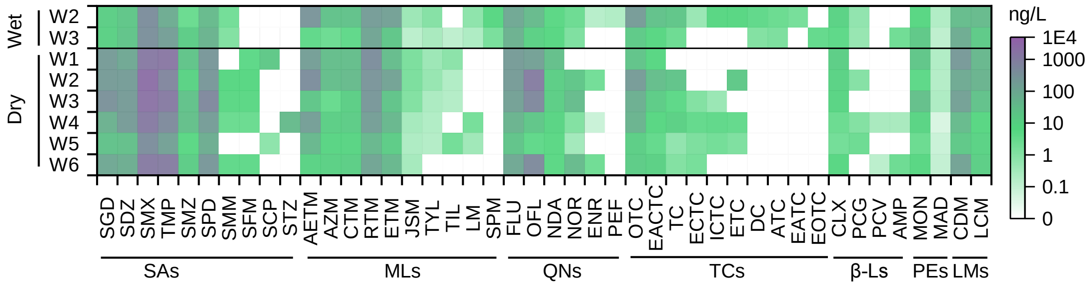

Figure S6. Antibiotic concentrations in E-STPs.

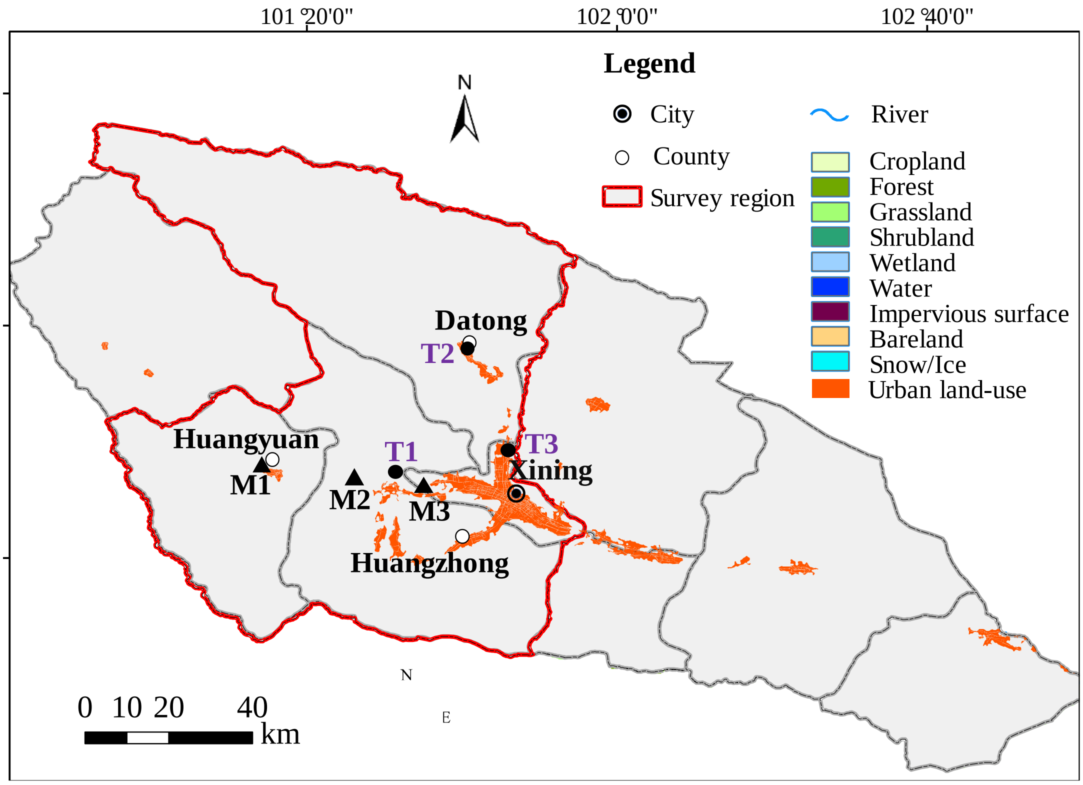

Figure S7. Land use of the Huangshui River basin. Map created using ArcGIS 10.2–http://developers.arcgis.com; Image from http://data.ess.tsinghua.edu.cn.

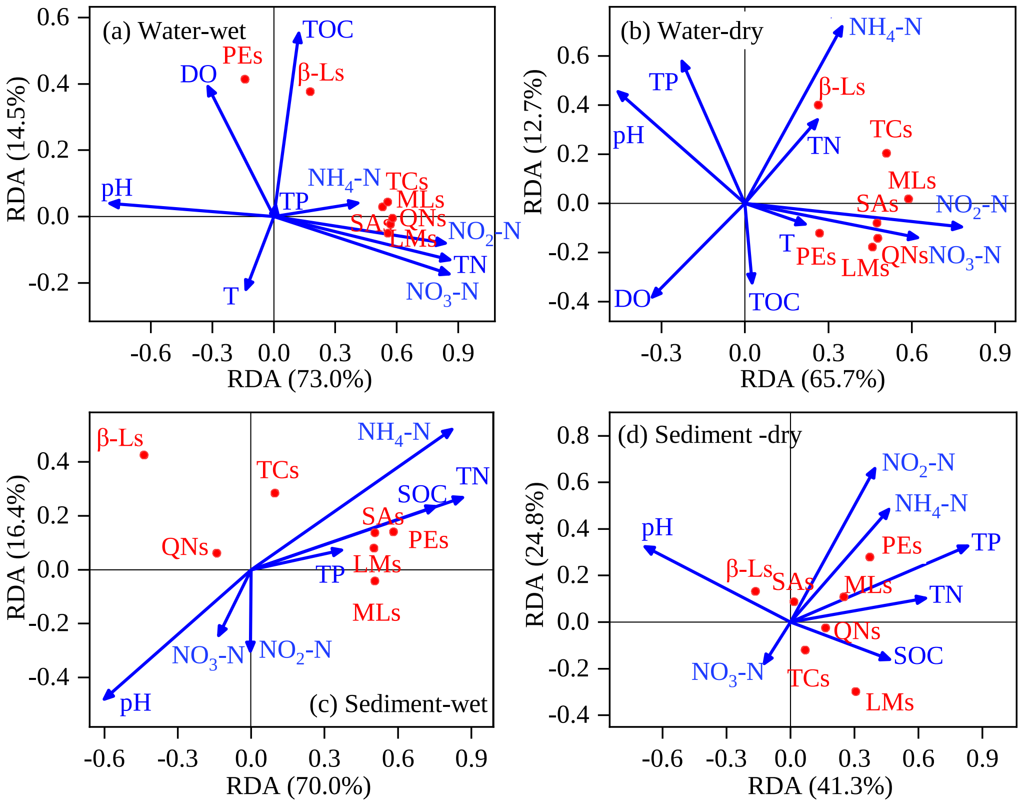

Figure S8. RDA analysis of different antibiotics in water (a and b) and sediments (c and d) from the Huangshui River.

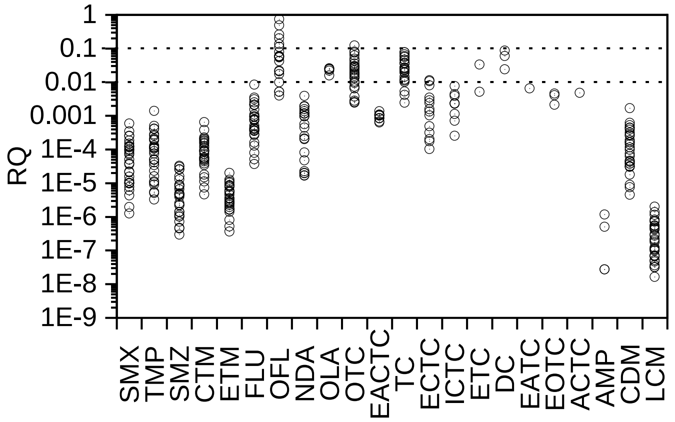

Figure S9. RQ values of antibiotics to bacteria in river water.

References

1. European. Technical Guidance Document in Support of Commission Directive 93/67/EEC on Risk Assessment for New Notified Substances, Commission Regulation (EC) No. 1488/94 on Risk Assessment for Existing Substance, and Directive 98/8/EC of the European Parliament and of the Council Concerning the Placing of Biocidal Products on the Market. Part II. European Commission Joint Research Centre 100–102 (EUR 20418 EN/2) (2003).
2. Li, S. et al. A duodecennial national synthesis of antibiotics in China's major rivers and seas (2005–2016). *Sci. Total Environ.* ***615,*** 906-917(2018).
3. Hernando, M.D., Mezcua, M., Fernandez-Alba, A.R. & Barcelo, D. Environmental risk assessment of pharmaceutical residues in wastewater effluents, surface waters and sediments. *Talanta* ***69,*** 334-342 (2006).
4. Chen, K. & Zhou, J.L. Occurrence and behavior of antibiotics in water and sediments from the Huangpu River, Shanghai, China. *Chemosphere* ***95,*** 604-612 (2014).
5. Qiang, Z.M. & Adams, C. Potentiometric determination of acid dissociation constants (p*K*a) for human and veterinary antibiotics. *Water Res.* ***38,*** 2874-2890 (2004).
6. Lin, C.E., Chang, C.C. & Lin, W.C. Migration behavior and separation of sulfonamides in capillary zone electrophoresis III. Citrate buffer as a background electrolyte. *J. Chromatogr. A* ***768,*** 105-112 (1997).
7. Geiser, L., Henchoz, Y., Galland, A., Carrupt, P.A. & Veuthey, J.L. Determination of pK(a) values by capillary zone electrophoresis with a dynamic coating procedure. J. Sep. Sci. ***28,*** 2374-2380 (2005).
8. Jimenez-Lozano, E., Marques, I., Barron, D., Beltran, J.L. & Barbosa, J. Determination of pK(a) values of quinolones from mobility and spectroscopic data obtained by capillary electrophoresis and a diode array detector. *Anal. Chim. Acta* ***464,*** 37-45 (2002).
9. McFarland, J.W. et al. Quantitative structure-activity relationships among macrolide antibacterial agents: In vitro and in vivo potency against Pasteurella multocida. *J. Med. Chem.* **40,** 1340-1346 (1997).
10. Zhao, H., Zhou, J.L. & Zhang, J. Tidal impact on the dynamic behavior of dissolved pharmaceuticals in the Yangtze estuary, China. *Sci. Total Environ.* ***536,*** 946-954 (2015).
11. Zhou, L., Wu, Q., Zhang, B., Zhao, Y. & Zhao, B. Occurrence, spatiotemporal distribution, mass balance and ecological risks of antibiotics in subtropical shallow Lake Taihu, China. *Environ. Sci. Processes Impacts* ***18,*** 500-513 (2016).
12. Ribeiro, A.R. & Schmidt, T.C. Determination of acid dissociation constants (pK(a)) of. cephalosporin antibiotics: Computational and experimental approaches. *Chemosphere* ***169,*** 524-533 (2017).
13. Kümmerer, K. & Henninger, A. (2003). Promoting resistance by the emission of antibiotics from hospitals and households into effluent. *Clin. Microbiol. Infect.* ***9,*** 1203-1214 (2003).
14. Bengtsson-Palme, J. & Larsson, D.G.J. Concentrations of antibiotics predicted to select for resistant bacteria: proposed limits for environmental regulation. *Environ. Int.* ***86,*** 140-149 (2016).
15. Zhou, H., Wu, C., Huang, X., Gao, M. & Tanaka, H. Occurrence of selected pharmaceuticals and caffeine in sewage treatment plants and receiving rivers in Beijing, China. *Water Environ. Res.* ***82(11),*** 2239-2248 (2010).

16. Ai, J., Hu, J., Wu, X., Peng, H., Wu, S. & Dong, Z. Occurrence and source apportionment of sulfonamides and their metabolites in Liaodong Bay and the adjacent Liao River basin, north China. *Environ. Toxicol. Chem.* ***30(6)***, 1252-1260 (2011).
17. Hu, W. The study on occurrence and distribution of typical pharmaceuticals and personal care products (PPCPs) in Tianjin urban aqueous and soil environment and the combined estrogenic effects. *Nankai University,* p. 119. In Chinese (2011).
18. Luo, Y., Xu, L., Rysz, M., Wang, Y., Zhang, H. & Alvarez, P.J.J. Occurrence and transport of tetracycline, sulfonamide, quinolone,and macrolide antibiotics in the Haihe River basin, China. *Environ. Sci. Technol.* ***45(5),*** 1827-1833 (2011).
19. Zou, S., Xu, W., Zhang, R., Tang, J., Chen, Y. & Zhang, G. Occurrence and distribution of antibiotics in coastal water of the Bohai Bay, China: Impacts of river discharge and aquaculture activities. *Environ. Pollut.* ***159,*** 2913-2920 (2011).
20. Gao, L., Shi, Y., Li, W., Liu, J. & Cai, Y. Occurrence, distribution and bioaccumulation of antibiotics in the Haihe River in China. *J. Environ. Monitor.* ***14,*** 1248–1255 (2012).
21. Yang, C., Wang, L., Hou, X. & Chen, J. Analysis of pollution levels of 16 antibiotics in the river water of Daliao River water system. *Chinese Journal of Chromatography* ***30(8),*** 756-762. In Chinese (2012).
22. Zhang, R. et al. Occurrence and risks of antibiotics in the Laizhou Bay, China: Impacts of river discharge. Ecotox. Environ. Safe. ***80,*** 208-215 (2012).
23. Zhang, Q. Determination and source apportionment of three classes of antibiotics in Beijing Wenyu Rivers. Chongqing University, p. 69. In Chinese (2012).
24. Zhang, R. et al. Occurrence and risks of antibiotics in the coastal aquatic environment of the Yellow Sea, north China. *Sci. Total Environ.* ***450,*** 197-204 (2013).
25. Wei, Y. Study on few typical antibiotics pollution characteristics and ecological toxicities in the river systems in the northern city, in China. *Liaoning University,* p. 78. In Chinese (2013).
26. Zhang, P. Distribution and health risk assessment on PPCPs in Hai River basin, China. *Central South University of Forestry and Technology*, p. 69. In Chinese (2013).
27. Gao, L., Li, X., Zhang, Y., Wei, Y., Li, W. & Feng, Z. Research on pollution characteristics of antibiotics in Qinghe River in Beijing. *Ecological Science* ***33,*** 83-92. In Chinese (2014).
28. Xu, Y. Pollution characteristics of antibiotics, antibiotic resistance bacteria and genes in Wangyanggou River, Shijiazhuang. *Shandong Agricultural University,* p. 70. In Chinese (2014).
29. Zhang, X. et al. Prevalence of veterinary antibiotics and antibiotic-resistant Escherichia coli in the surface water of a livestock production region in Northern China. *Plos One,* **9,** e111026 (2014).
30. Bai, Y., Meng, W., Xu, J., Zhang, Y. & Guo, C. Occurrence, distribution and bioaccumulation of antibiotics in the Liao River basin in China. *Environ. Sci.-Process Impacts* ***16(3),*** 586-593 (2014).
31. Jiang, Y. et al. Distribution and ecological risk of antibiotics in a typical effluent-receiving river (Wangyang River) in north china. *Chemosphere* ***112,*** 267-274 (2014).
32. Li, W., Gao, L., Shi, Y., Liu, J. & Cai, Y. Occurrence, distribution and risks of antibiotics in urban surface water in Beijing, China. *Environ. Sci.-Process Impacts* ***17(9),*** 1611-1619 (2015).

33. Qin, Y., Zhang, L., Shi, Y., Ma, Y., Chang, X. & Liu, Z. Contamination characteristics and ecological risk assessment of typical antibiotics in surface water of the Daliao River, China. *Research of Environmental Sciences* ***28(3),*** 361-368. In Chinese (2015).
34. Wang, Z., Zhang, X.H., Huang, Y. & Wang, H. Comprehensive evaluation of pharmaceuticals and personal care products (PPCPs) in typical highly urbanized regions across China. *Environ. Pollut.* ***204,*** 223-232 (2015).
35. Li, Q., Gao, J., Zhang, Q., Liang, L. & Tao, H. Distribution and risk assessment of antibiotics in a typical river in north china plain. *Bull. Environ. Contam. Toxicol.* ***98(4),*** 478-483 (2017).
36. Ma, R. et al. Characterization of pharmaceutically active compounds in Beijing, China: occurrence pattern, spatiotemporal distribution and its environmental implication. *J. Hazard. Mater.* ***323,*** 147-155 (2017).
37. Ran, Y. Distribution of 13 polar PPCPs in a river basin of Beijing-Tianjin-Hebei and modeling transformation of PPCP under denitrifying conditions. *Hebei University of Technology*, p.74. In Chinese (2017).
38. Yan, X. Distribution, source and risk assessment of typical antibiotics in Xiaoqing river basin. *Xi'an University of Technology,* p.84. In Chinese (2018).
39. Liu, X. et al. Occurrence and fate of antibiotics and antibiotic resistance genes in typical urban water of Beijing, China. *Environ. Pollut.* ***246,*** 163-173 (2018).
40. Lei, K., Zhu, Y., Chen, W., Pan, H. & Liu, X. Spatial and seasonal variations of antibiotics in river waters in the haihe river catchment in china and ecotoxicological risk assessment. Environ. Int. ***130,*** 104919 (2019).
41. Sun, Y. Pollution characteristics of antibiotics and antibiotic resistance genes in urban water environment. *Hebei University of Engineering* p. 83. In Chinese (2019).
42. Zhuo, X. Determination of fluoquinolones by fluorimetry and its residues analysis in the water environment. *Zhejiang University,* p.69. In Chinese (2007).
43. Zhang, C., Hu, G. & Sun, C. Simultaneous analysis of 7 antibiotics in water by ultra performance liquid chromatography-electrospray ionization-tandem mass spectrometry. *The Administration and Technique of Environmental Monitoring* **21,** 37-40. In Chinese (2009).
44. Jiang, L., Hu, X., Yin, D., Zhang, H. & Yu, Z. Occurrence, distribution and seasonal variation of antibiotics in the Huangpu River Shanghai China. *Chemosphere,* ***82(6),*** 822-828 (2011).
45. Shen, Q., Ji, X., Fu, S., Liu, Y. & Li, L. Preliminary studies on the pollution levels of antibiotic and antibiotic resistance genes in Huangpu River, China. *Ecology and Environmental Sciences* ***21(10),*** 1717-1723. In Chinese (2012).
46. Jiang, L., Hu, X., Xu, T., Zhang, H., Sheng, D. & Yin, D. Prevalence of antibiotic resistance genes and their relationship with antibiotics in the Huangpu River and the drinking water sources, Shanghai, China. *Sci. Total Environ.* ***458,*** 267-272 (2013).
47. Yan, Q. et al. Pollution level and ecological risk assessment of typical pharmaceutically active compounds in the river basins of main districts of Chongqing. *Research of Environmental Sciences* ***26(11),*** 1178-1185. In Chinese (2013).
48. Shi, X., Zhou, J.L., Zhao, H., Hou, L. & Yang, Y. Application of passive sampling in assessing the occurrence and risk of antibiotics and endocrine disrupting chemicals in the Yangtze estuary, China. *Chemosphere* ***111,*** 344-351 (2014).

49. Wu, C. et al. Occurrence of pharmaceuticals and personal care products and associated environmental risks in the central and lower Yangtze River, China. *Ecotox. Environ. Safe.* ***106,*** 19-26 (2014).
50. Xu, H. Determination of antibiotics in Shanghai and the study of its environmental behavior. *Shanghai University,* p.88. In Chinese (2015).
51. Xu, H., Wu, M. & Xu, G. Analytical methods of 12 antibiotics in aqueous environment. *Journal of Shanghai University (Natural Science)* ***22,*** 1-9. In Chinese (2016).
52. Zhao, H. Environmental behavior of typical pharmaceutical residues in the Yangtze Estuary and optimization of monitoring technology. *East China Normal University,* p. 147. In Chinese (2016).
53. Fang, L. Pollution characteristics and ecological risk assessment of typical antibiaotics in the upper Huangpu River. *Donghua University,* p. 69. In Chinese (2016).
54. Jin, L. et al. Distribution characteristics and health risk assessment of thirteen sulfonamides antibiotics in a drinking water source in East China. *Environmental Science* ***37(7),*** 2515-2521. In Chinese (2016).
55. Zong, Y., Shao, M., Liang, M., Tang, J. & Wang, R. Occurrence and distribution of antibiotics in the surface water of a typical urban river in the Yangtze River Delta. *Journal of Agro-Environment Science* ***37,*** 965-973. In Chinese (2018).
56. Guo, X., Xiaojun, L., Zhang, A., Yan, Z., Chen, S. & Wang, N. Antibiotic contamination in a typical water-rich city in southeast china: a concern for drinking water resource safety. *J. Environ. Sci. Health Part B-Pestic. Contam. Agric. Wastes* ***55(3),*** 193-209 (2019).
57. Pan, C., Bao, Y. & Xu, B. Seasonal variation of antibiotics in surface water of Pudong New Area of Shanghai, China and the occurrence in typical wastewater sources. *Chemosphere* ***239,*** 124816 (2020).
58. Tan, J., Tang, C., Yu, Y. & Peng, X. Simultaneous analysis of multiple classes of antibiotics in urban river water by high performance liquid chromatography. *Chinese Journal of Chromatography* ***25(4),*** 546-549. In Chinese (2007).
59. Xu, W. Occurrence and environmental fate of selected antibiotics in the aquatic environment of the Pearl River Delta. *Graduate university of Chinese academy of sciences (Guangzhou institute of geochemistry),* p.123. In Chinese (2007).
60. Ye, J., Zou, S., Zhang, G. & Xu, W. Characteristics of selected antibiotics in the aquatic environment of the Pearl River Delta, south China. *Ecology and Environment,* ***16,*** 384-388. In Chinese (2007).
61. Peng, X., Tan, J., Tang, C., Yu, Y. & Wang, Z. Multiresidue determination of fluoroquinolone, sulfonamide, trimethoprim, and chloramphenicol antibiotics in urban waters in China. *Environ. Toxicol. Chem.* ***27(1),*** 73-79 (2008).
62. Yang, J., Ying, G., Zhao, J., Tao, R., Su, H. & Liu, Y. Spatial and seasonal distribution of selected antibiotics in surface waters of the Pearl Rivers, China. *J. Environ. Sci. Health Part B-Pestic. Contam. Agric. Wastes* ***46(3),*** 272-280 (2011).
63. Zhang, D., Lin, L., Luo, Z., Yan, C. & Zhang, X. Occurrence of selected antibiotics in Jiulongjiang River in various seasons, South China. *J. Environ. Monitor.* ***13(7),*** 1953-1960 (2011).
64. Zhang, R. et al. Levels, spatial distribution and sources of selected antibiotics in the East River (Dongjiang), South China. *Aquat. Ecosyst. Health Manag.* ***15,*** 210-218 (2012).

65. Zhang, X., Zhang, D., Zhang, H., Luo, Z. & Yan, C. Occurrence, distribution, and seasonal variation of estrogenic compounds and antibiotic residues in Jiulongjiang River, South China. *Environ. Sci. Pollut. Res.* ***19,*** 1392-1404 (2012).
66. Jiang, H., Zhang, D., Xiao, S., Geng, C. & Zhang, X. Occurrence and sources of antibiotics and their metabolites in river water, WWTPs, and swine wastewater in Jiulongjiang River basin, south China. *Environ. Sci. Pollut. Res.* ***20(12),*** 9075-9083 (2013).
67. Chen, B., Liang, X., Nie, X., Huang, X., Zou, S. & Li, X. The role of class I integrons in the dissemination of sulfonamide resistance genes in the Pearl River and Pearl River Estuary, South China. *J. Hazard. Mater.* ***282,*** 61-67 (2015).
68. Jiang, H. et al. Occurrence and potential sources of antibiotics in Beijiang River, Southern China. *Asian Journal of Ecotoxicology* ***10(5),*** 132-140. In Chinese (2015).
69. Ou, D., Chen, B., Bai, R., Song, P. & Lin, H. Contamination of sulfonamide antibiotics and sulfamethazine-resistant bacteria in the downstream and estuarine areas of Jiulong River in Southeast China. *Environ. Sci. Pollut. Res.* ***22,*** 12104-12113 (2015).
70. Huang, Q., Chen, Q., Lei, M., Wei, G. & Chou, T. Simultaneous determination of trace antibiotics in surface water by isotopediluted high performance liquid chromatography-mass spectrometry. *Environ. Chem.* ***35,*** 1493-1499. In Chinese (2016).
71. Zhang, R., Zhang, R., Li, J., Cheng, Z. & Zhang, G. Occurrence and distribution of antibiotics in multiple environmental media of the East River (Dongjiang) catchment, South China. *Environ. Sci. Pollut. Res*. **24(10),** 1-12 (2017).
72. Li, S. et al. Antibiotics in water and sediments of rivers and coastal area of Zhuhai City, Pearl River estuary, south China. *Sci. Total Environ.* ***636,*** 1009-1019(2018).
73. Wei, X. Contamination of antibiotics in typical emission sources and a river in Guangzhou. *University of Chinese Academy of Sciences,* p.101. In Chinese (2018).
74. Huang, Y.H. et al. Occurrence and distribution of antibiotics and antibiotic resistant genes in water and sediments of urban rivers with black-odor water in Guangzhou, South China. *Sci. Total Environ.* ***670,*** 170-180 (2019).
75. Qiu, W. et al. Occurrence of antibiotics in the main rivers of Shenzhen, China: Association with antibiotic resistance genes and microbial community. *Sci. Total Environ.* ***653,*** 334-341 (2019).
76. Li K., Li X., Qiu F., Zhou Z. & Liang Z. Determination and distribution characteristics of 20 antibiotics in major rivers and bays of Shenzhen. *Journal of environmental hygiene*, ***9(05),*** 455-461. In Chinese (2019).
77. Zhou, Z., Li, X., Li, K., Xu, Y., Zhao, L. & Chen, S., 2019. Investigation and source analysis of antibiotic pollution in major rivers in Shenzhen. *Modern Preventive Medicine* ***46,*** 3120-3125. In Chinese (2019).
78. He, S. et al. Occurrence and ecological risk assessment of 22 emerging contaminants in the Jilin Songhua River (northeast China). *Environ. Sci. Pollut. Res.* ***25,*** 24003–24012 (2018).
79. Wang, J. Distribution characteristics and ecological risk assessment of antibiotics in surface waters of xi 'an section of Weihe River. *Xi'an University of Technology,* p.80. In Chinese (2018).
80. He, S. et al. Contaminants of emerging concern in a freeze-thaw river during the spring flood. *Sci. Total Environ.* **670,** 576-584 (2019).
81. Yu, Y., Wu, G., Wang, C., Lu, N. & Zhu, X. Pollution characteristics of antibiotics and antibiotic resistance of coliform bacteria in the Yitong River, China. *Environ. Monit. Assess.* ***191(8),*** 516 (2019).

82. Liu, H., Zhang, G. & Liu, C. Determination of chloramphenicol and three tetracyclines by solid phase extraction and high performance liquid chromatography-ultraviolet detection. *Chinese Journal of Analytical Chemistry* ***35(3),*** 315-319. In Chinese (2007).
83. Liu, H., Zhang, G., Liu, C.Q., Li, L. & Xiang, M. The occurrence of chloramphenicol and tetracyclines in municipal sewage and the Nanming River, Guiyang City, China. *J. Environ. Monitor.* ***11(6),*** 1199-1205 (2009).
84. Xu, W., Zhang, G., Zou, S., Ling, Z., Wang, G. & Yan, W. A preliminary investigation on the occurrence and distribution of antibiotics in the Yellow River and its tributaries, China. *Water Environ. Res.* ***81(3),*** 248-254 (2009).
85. Chang, X. et al. Determination of antibiotics in sewage from hospitals, nursery and slaughter house, wastewater treatment plant and source water in Chongqing region of Three Gorge Reservoir in China. *Environ. Pollut.* ***158(5),*** 1444-1450 (2010).
86. Yan, C. et al. Antibiotics in the surface water of the Yangtze estuary: occurrence, distribution and risk assessment. *Environ. Pollut.* ***175(Apr.),*** 22-29 (2013).
87. Xue, B. et al. Occurrence, distribution and ecological risks of sulfonamides in the Qinzhou Bay, South China. China *Environmental Science* **33,** 1664-1669. In Chinese (2013).
88. Xue, B., Zhang, R., Wang, Y., Liu, X., Li, J. & Zhang, G. (2013b). Antibiotic contamination in a typical developing city in south China: Occurrence and ecological risks in the Yongjiang River impacted by tributary discharge and anthropogenic activities. *Ecotox. Environ. Safe.* ***92,*** 229-236 (2013).
89. Yao, L. et al. Occurrence and risk assessment of antibiotics in surface water and groundwater from different depths of aquifers: a case study at Jianghan plain, Central China. *Ecotox. Environ. Safe.* ***135(JAN.),*** 236-242 (2017).
90. Jia, J. et al. Occurrence and distribution of antibiotics and antibiotic resistance genes in Ba River, China. *Sci. Total Environ.* ***642,*** 1136-1144 (2018).
91. Wang, Y. et al. Distribution characteristics of typical antibiotics in urban rivers of Guiyang City. *Environmental Chemistry* ***37,*** 2039-2048. In Chinese (2018).
92. Yang, Y. Concentration levels and distribution characteristics of various antibiotics in Kaidu River and Kongque River in Bazhou area, Xinjiang. *Shihezi University,* p.66. In Chinese (2018).
93. Wang, J., Wei, H., Zhou, X., Li, K., Wu, W. & Guo, M. Occurrence and risk assessment of antibiotics in the Xi'an section of the Weihe River, northwestern China. *Mar. Pollut. Bull.* ***146,*** 794-800 (2019).
94. Murata, A., Takada, H., Mutoh, K., Hosoda, H., Harada, A., Nakada, N. Nationwide monitoring of selected antibiotics: distribution and sources of sulfonamides, trimethoprim, and macrolides in Japanese rivers. *Sci. Total Environ.* ***409***, 5305-5312 (2011).
95. Tran, N.H. et al. Occurrence and risk assessment of multiple classes of antibiotics in urban canals and lakes in Hanoi, Vietnam. *Sci. Total Environ.* ***692***, 157-174 (2019).
96. Böger, B. et al. Occurrence of antibiotics and antibiotic resistant bacteria in subtropical urban rivers in Brazil. *J. Hazard. Mater.* ***402***, 123448 (2021).
97. Batt, A.L. Kincaid, T.M., Kostich, M.S., Lazorchak, J.M. & Olsen, A,R. Evaluating the extent of pharmaceuticals in surface waters of the United States using a national-scale rivers and streams assessment survey. *Environ. Toxicol. Chem.* ***35(4)***, 874-881 (2016).

98. Watkinson, A.J., Murby, E.J., Kolpin, D.W., Costanzo, S.D. The occurrence of antibiotics in an urban watershed: From wastewater to drinking water. *Sci. Total Environ.* ***407***, 2711-2723 (2009).
99. Bialk-Bielinska, A. et al. Ecotoxicity evaluation of selected sulfonamides. *Chemosphere* 85, 928-933 (2011).
100. Ortiz de Garcia, S. A., Pinto Pinto, G., Garcia-Encina, P. A. & Irusta-Mata, R. Ecotoxicity and environmental risk assessment of pharmaceuticals and personal care products in aquatic environments and wastewater treatment plants. *Ecotoxicology* 23, 1517-1533 (2014).
101. Kim, Y. et al. Aquatic toxicity of acetaminophen, carbamazepine, cimetidine, diltiazem and six major sulfonamides, and their potential ecological risks in Korea. *Environ. Int.* 33, 370-375 (2007).
102. Isidori, M., Lavorgna, M., Nardelli, A., Pascarella, L. & Parrella, A. Toxic and genotoxic evaluation of six antibiotics on non-target organisms. *Sci. Total Environ.* 346, 87-98 (2005).
103. Majewsky, M. et al. Antibacterial activity of sulfamethoxazole transformation products (TPs): General relevance for sulfonamide TPs modified at the para position. *Chem. Res. Toxicol.* 27, 1821-1828 (2014).
104. Ferrari, B. et al. Environmental risk assessment of six human pharmaceuticals: Are the current environmental risk assessment procedures sufficient for the protection of the aquatic environment? *Environ. Toxicol. Chem.* 23, 1344-1354 (2004).
105. Ioele, G., De Luca, M. & Ragno, G. Acute toxicity of antibiotics in surface waters by bioluminescence test. *Curr. Pharm. Anal.* 12, 220-226 (2016).
106. Choi, K. et al. Occurrences and ecological risks of roxithromycin, trimethoprim, and chloramphenicol in the Han River, Korea. *Environ. Toxicol. Chem.* 27, 711-719 (2008).
107. Zounková, R., Klimešová, Z., Nepejchalová, L., Hilscherová, K. & Bláha, L. Complex evaluation of ecotoxicity and genotoxicity of antimicrobials oxytetracycline and flumequine used in aquaculture. *Environ. Toxicol. Chem.* 30, 1184-1189 (2011).
108. Lalumera, G. M. et al. Preliminary investigation on the environmental occurrence and effects of antibiotics used in aquaculture in Italy. *Chemosphere* 54, 661-668 (2004).
109. Backhaus, T., Scholze, M. & Grimme, L. H. The single substance and mixture toxicity of quinolones to the bioluminescent bacterium *Vibrio fischeri*. *Aquat. Toxicol.* 49, 49-61 (2000).
110. Park, S. & Choi, K. Hazard assessment of commonly used agricultural antibiotics on aquatic ecosystems. *Ecotoxicology* 17, 526-538 (2008).
111. Yuan, F. et al. Photodegradation and toxicity changes of antibiotics in UV and UV/H_2O_2 process. *J. Hazard. Mater.* 185, 1256-1263 (2011).
112. Kołodziejska, M. et al. Aquatic toxicity of four veterinary drugs commonly applied in fish farming and animal husbandry. *Chemosphere* 92, 1253-1259 (2013).
113. Suda, T., Hata, T., Kawai, S., Okamura, H. & Nishida, T. Treatment of tetracycline antibiotics by laccase in the presence of 1-hydroxybenzotriazole. *Bioresour. Technol.* 103, 498-501 (2012).
114. Backhaus, T. & Grimme, L. H. The toxicity of antibiotic agents to the luminescent bacterium *Vibrio fischeri*. *Chemosphere* 38, 3291-3301 (1999).

115. Ji, J.Y., Xing, Y.J., Ma, Z.T., Zhang, M. & Zheng, P. Acute toxicity of pharmaceutical wastewaters containing antibiotics to anaerobic digestion treatment. *Chemosphere* 91, 1094-1098 (2013).
